# Combinatory optimization of chromosomal integrated mevalonate pathway for β-carotene production in *Escherichia coli*

**DOI:** 10.1186/s12934-016-0607-3

**Published:** 2016-12-01

**Authors:** Lijun Ye, Chunzhi Zhang, Changhao Bi, Qingyan Li, Xueli Zhang

**Affiliations:** 1 School of Biological Engineering, Dalian Polytechnic University, Dalian, 116034 People’s Republic of China; 2 Tianjin Institute of Industrial Biotechnology, Chinese Academy of Sciences, Tianjin, 300308 People’s Republic of China; 3 Key Laboratory of Systems Microbial Biotechnology, Chinese Academy of Sciences, Tianjin, 300308 People’s Republic of China

**Keywords:** *Escherichia coli*, Isoprenoid, β-carotene, MVA pathway, RBS

## Abstract

**Background:**

Plasmid expression is a popular method in studies of MVA pathway for isoprenoid production in *Escherichia coli*. However, heterologous gene expression with plasmid is often not stable and might burden growth of host cells, decreases cell mass and product yield. In this study, MVA pathway was divided into three modules, and two heterologous modules were integrated into the *E. coli* chromosome. These modules were individually modulated with regulatory parts to optimize efficiency of the pathway in terms of downstream isoprenoid production.

**Results:**

MVA pathway modules *Hmg1*-*erg12* operon and *mvaS*-*mvaA*-*mavD1* operon were integrated into *E. coli* chromosome followed by modulation with promoters with varied strength. Along with activation of *atoB*, a 26% increase of β-carotene production with no effect on cell growth was obtained. With a combinatory modulation of two key enzymes *mvas* and *Hmg1* with degenerate RBS library, β-carotene showed a further increase of 51%.

**Conclusions:**

Our study provides a novel strategy for improving production of a target compound through integration and modulation of heterologous pathways in both transcription and translation level. In addition, a genetically hard-coded chassis with both efficient MEP and MVA pathways for isoprenoid precursor supply was constructed in this work.

**Electronic supplementary material:**

The online version of this article (doi:10.1186/s12934-016-0607-3) contains supplementary material, which is available to authorized users.

## Background

Isoprenoids, also referred to as terpenes or terpenoids, are the most diverse class of natural products consisting of over 55,000 structurally different compounds, which has lots of applications in pharmaceuticals, nutraceuticals, cosmetics and food [1–4]. These valuable compounds are commonly isolated from plant, microbes and marine organisms. But the supply of these compounds has been limited by scarce resources from which they were originally extracted. Production by chemical synthesis is uneconomical due to the complex structure of these products [1]. For these reasons, microbial metabolic engineering has been explored in the past decade for isoprenoid production, including artemisinin, limonene paclitaxel (Taxol), astaxanthin, β-carotene and lycopene etc. [5–8].

Isoprenoids are all derived from two five-carbon building blocks called isopentenyl diphosphate (IPP) and dimethylallyl diphosphate (DMAPP), which are synthesized either by the mevalonate (MVA) pathway in eukaryotes, archaea, and some bacteria or 2-C-methyl-d-erythritol-4-phosphate (MEP) pathway in other prokaryotes and plastids in plants [2, 3, 9]. In the past 15 years, to increase the yield of terpene production, considerable effort has been focused on improving precursor supply by overexpression or deletion of upstream pathway genes, and altering global metabolic network by rational strategies or random mutagenesis [10–12]. MEP pathway has been engineered to increase IPP and DMAPP in *E. coli* for increased synthesis of carotenoids. Yuan et al. showed that four enzymes in the MEP pathway were rate limiting [13]. Similarly, when intrinsic *dxs* and *idi* were modulated by artificial modulation parts, the resultant strains had increased β-carotene production [14]. On the other hand, to address precursor IPP/DMAPP limitations in *E. coli*, heterologous MVA pathway genes were overexpressed using plasmid to improve isoprenoid production [15–19]. Higher isoprenoid production were achieved by strains equiped with the bottom portion of MVA pathway of *Streptococcus pneumoniae*, and cultured in media with MVA supplementation [18, 19]. Lycopene production of *E. coli* harboring the whole MVA pathway from *Streptomyces* sp. CL190 was two-fold higher than strain with only native MEP pathway [16]. However, high-level expression of mevalonate pathway enzymes might inhibit cell growth. Pitera et. al found that accumulation of MVA pathway intermediate 3-hydroxy-3-methyl-glutaryl-coenzyme A (HMG-CoA) inhibited cell growth with overexpressed *atoB*, *mvaS* and *hmg1* [20]. Mevalonate kinase (MK), encoded by *erg12,* was identified as another rate-limiting enzyme when the MVA pathway was used to increase in amorphadiene production [21].

**Fig. 1:**
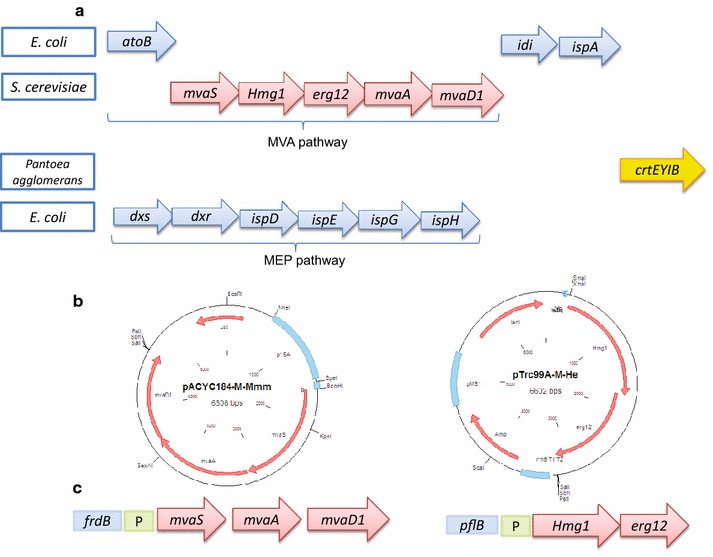
Genes used for β-carotene synthesis in engineered *E. coli* strains, vector constructs and the two artificial MVA operons. **a** Genes involved in β-carotene Production via both MEP and MVA pathways. The abbreviations for enzymes and pathway intermediates are as follows: *atoB*, gene of acetoacetyl-CoA thiolase; *mvaS*, gene of HMG-CoA synthase; *Hmg1*, truncated HMG-CoA reductase; *erg12*, gene of mevalonate kinase; mvaA, gene of HMG-CoA reductase; *mvaD1*, gene of mevalonate pyrophosphate decarboxylase; *idi*, gene of IPP isomerase; *ispA*, FPP synthase; *crtEYIB*, β-carotene synthesis operon from *Pantoea agglomerans*; **b** Plasmid maps of vectors with heterologous MVA genes; **c** Integrated artificial MVA pathway operons in *E. coli* chromosome at *pflB* and *frdB* sites

To balance MVA pathway flux, it is necessary to express the HMG-CoA reductase and MK at a higher level to decrease accumulation of HMG-CoA, and to eliminate the rate-limiting step. In our previous work, β-carotene synthetic gene operon (*crtEXYIB*) from *P. agglomerans* CGMCC No. 1.2244 controlled by trc promoter and rrnB transcriptional terminator was integrated into wild type *E. coli* ATCC 8739 at *ldhA* site, resulting in strain QL002. The inducible promoter of *crtEXYIB* in QL002 was replaced with strong constitutive promoter M1-12 to obtain strain QL105. Activation of *dxs*, *idi* genes and *crt* operon in QL105 led to increase of β-carotene production, and the resulting stain was named CAR001 [14]. In this study, the MVA pathway genes were divided into three modules, (i) *hmg1* and *erg12*, which need to be expressed at high level, (ii) *atoB*, which is an endogenous gene of *E. coli*, and (iii) *mvaS*, *mvaA* and *mvaD1*, which are the other genes of the MVA pathway (Fig. 1a). The objective of the study was to increase β-carotene production by integrating heterologous genes of MVA pathway into *E. coli* chromosome as two operons, modulate involved heterologous and endogenous genes individually, as well as illustrate relationship between gene expression level and β-carotene production in hyper producer strain.

## Methods

### Strains, medium and growth conditions

Strains used in this study are listed in Additional file 1: Table S6. During strain construction, cultures were grown aerobically at 30, 37, or 39 °C in Luria broth (per liter: 10 g Difco tryptone, 5 g Difco yeast extract and 5 g NaCl). For β-carotene production, single colonies were picked from the plate and inoculated into 15 × 100 mm tubes containing 4 ml LB with or without 34 mg/l chloramphenicol and 100 mg/l ampicillin, and grown at 30 °C and 250 rpm overnight. Seed culture was then inoculated into 100 ml flask containing 10 ml LB, with or without 34 mg/l chloramphenicol and 100 mg/l ampicillin (with an initial OD_600_ of 0.05), and grown at 30 °C and 250 rpm. After 24 h growth, cells were collected for measurement of β-carotene production. For strains using trc promoter for induction of MVA pathway genes, 1 mM IPTG was added for induction 3 h after inoculation, followed by 21 h growth [14].

### Construction of plasmids for expressing MVA pathway genes

All plasmids used in this study are listed in Additional file 1: Table S5. The *Hmg1* and *erg12* genes, which need be expressed at high level, were placed in one operon; while *mvas*, *mvaA* and *mavD1* genes were put in another operon (Fig. 1a). *Hmg1* and *erg12* were isolated by PCR with Pfu DNA polymerase (NEB) from chromosomal DNA of *Saccharomyces cerevisiae*. Individual genes were spliced together (sequence named as He) using overlapping extensions from primers HMG1-XmaI-f, HMG1-r, ERG12-f, ERG12-SalI-r (Additional file 1: Table S1). *mvas*, *mvaA* and *mavD1* genes were isolated and spliced together (sequence named as Mmm) by overlapping extensions from primers ERG13-BamHI-f, ERG13-r, ERG8-f, ERG8-r, MVD1-f and MVD1-SalI-r (Additional file 1: Table S1). Plasmid pTrc99A-M were digested by *Xma*I and *Sal*I ligated by T4 DNA ligase, and transformed into Trans T1 competent cells (Transgen, Beijing, CN). Plasmid carrying *Hmg1* and *erg12* genes was screened, selected and designated as pTrc99A-M-He (Fig. 1b). *mvas, mvaA* and *mavD1* genes were inserted into pACYC184-M at *Bam*HI and *Sal*I site using the same method, and the plasmid was designated as pACYC184-M-Mmm.

### Integration of MVA genes into *E. coli* chromosome

A two-step homologous recombination method [22, 23] was used to integrate *Hmg1*-*erg12* operon into *E. coli* CAR001 [14] at *pflB* site, and the *mvaS*-*mvaA*-*mavD1* operon at *frdB* site (Fig. 1c). *pflB* gene was amplified from genomic DNA of *E. coli* ATCC 8739 using primer set pflB-up/pflB-down (Additional file 1: Table S1), and cloned into pEASY-Blunt (Transgen, Beijing, CN) to produce plasmid pXZ014 (Additional file 1: Table S5). A 1000-fold dilution of this plasmid DNA served as template for inside-out amplification using the pflB-1/pflB-2 primer set (Additional file 1: Table S1). The resulting 4735 bp fragment containing replicon was ligated with *cat*-*sacB* cassette from pXZ-CS [24] to produce pXZ015C (Additional file 1: Table S5). PCR fragment amplified from pXZ014 was ligated with the *Hmg1*-*erg12* operon, which was amplified from pTrc99A-M-He with prime set HMG1-XmaI-f/99A-r (Additional file 1: Table S1), to produce plasmid pQL003-He (Additional file 1: Table S5). A two-step recombination method was developed for markerless recombination of *Hmg1*-*erg12* operon expression in CAR001 at *pflB* site (Additional file 1: Figure S1). In the first recombination, *cat*-*sacB* cassette was amplified from pXZ015C with primer set pflB-up/pflB-down (Additional file 1: Table S1), treated with *Dpn*I, and electroporated into competent CAR001 with pKD46. After overnight growth on LB plate with 34 mg/l chloramphenicol and 100 mg/l ampicillin at 30 °C, several colonies were picked for PCR verification using primer set cat-up/pflB-down (Additional file 1: Table S1). In the second recombination, *Hmg1*-*erg12* operon with rrnB terminator were amplified from pQL003-He with a same primer set pflB-up/pflB-down (Additional file 1: Table S1), and used to replace *cat*-*sacB* cassette by selection for resistance to sucrose. Cells containing *sacB* gene accumulated levan during incubation with sucrose and were eliminated. With this mechanism, survived recombinants were highly enriched for colonies without *cat*-*sacB* cassette [22, 23]. The resulting strain was designated as CAR006. Plasmid pQL006-Mmm was constructed using the same method as pQL003-He. *mvaS*-*mvaA*-*mavD1* operon was inserted into *frdB* site using the same method as integration of *Hmg1*-*erg12* operon. The primers used are listed in Additional file 1: Table S1. Plasmids are listed in Additional file 1: Table S5.

### Two-step recombination method for markerless modulation of gene expression

A two-step recombination method was used for markerless modulation of gene expression, which was beneficial for multiple rounds of genome editing [25]. *Hmg1*-*erg12* operon was first modulated in CAR006 using this method (Additional file 1: Figure S2). In the first recombination, *cat*-*sacB* cassette was amplified with primer set pflB-up-cat/Hmg1-sacB-down (Additional file 1: Table S1) for insertion at upstream of *Hmg1*-*erg12* operon. In the second recombination, different artificial regulatory parts (M1-46 and M1-93, whose strengths were 2.5 and 5 times of induced *E. coli lacZ* when cultivated in LB medium [26]) were amplified with a same primer set pflB-up-P/Hmg1-RBS-down (Additional file 1: Table S1) and used to replace *cat*-*sacB* cassette by selection for resistance to sucrose. Markerless modulation of other genes was the same as *Hmg1*-*erg12* operon, and primers used are listed in Additional file 1: Table S1. Resulting strains are listed in Additional file 1: Table S6.

### One-step recombination method for modulating *mvas* and *Hmg1* expression with RBS library

*mvas* of CAR012 was modulated with RBS library using a one-step recombination method as described previously [26, 27]. Regulatory parts M1-93 was PCR template for RBS library. The artificial regulatory parts with different resistance gene were constructed to be used as templates for RBS library construction, so that different genes can be modulated in a one strain with resistance genes intact. For construction of M1-cam-93, a chloramphenicol resistance gene fragment followed by 50 nucleotides homologous sequence of FRT in M1-93 was amplified from plasmid pXZ-CS using primer set FRT-cam-up/FRT-cam-down (Additional file 1: Table S1), and electroporated into competent M1-93 with pKD46. After overnight growth on LB plate with 34 mg/l chloramphenicol, several colonies were picked for PCR verification using primer set Cat-g-up/LacZ-373 (Additional file 1: Table S1). The obtained strain was designated M1-cam-93, as listed in Additional file 1: Table S6.

Apramycin resistance regulatory parts construction was the same as M1-cam-93, primers are listed in Additional file 1: Table S1, and the resultant strain was designated as M1-Apr-93 (Additional file 1: Table S6).

A one-step homologous recombination method was used to further modulate *mvas* and *Hmg1* gene with RBS Library. For modulating *mvas*, DNA fragments were amplified from genomic DNA of M1-cam-93 with primer set frdB-up-FRT/Mvas-RBSL-down (Additional file 1: Table S2). Primer Mvas-RBSL-down was degenerated with six bp random sequence at RBS site (NNNNNNYC) [28]. The resulting PCR product was a degenerated RBS library, and electroporated into competent *E. coli* CAR012 with pKD46. After overnight growth on LB plate with 34 mg/l chloramphenicol, fifteen colonies were picked for PCR verification using primer set Cat-g-up/MvaS-350-r (Additional file 1: Table S1). Fifteen correct colonies were randomly selected and designated from mvaS-RBSL-1 to mvaS-RBSL-15 (Additional file 1: Table S6), β-carotene production of which were measured (Additional file 1: Table S6). *Hmg1* was modulated with RBS Library in the same way of the *mvas*, except that the RBS library was PCR amplified from M1-Apr-93 with apramycin resistance gene. Primers are listed in Additional file 1: Table S1 and strains are listed in Additional file 1: Table S6.

To further increase the β-carotene production, *Hmg1* gene of the *Hmg1*-*erg12* operon in the best strain mvaS-RBSL-13 was also modulated with the RBS Library. In addition, a ten-colony-strain mixture randomly selected from mvaS-RBS Library was also used as parent strain pool for *Hmg1* modulation. Fifteen colonies from each resulted library were randomly selected for measuring β-carotene production (Additional file 1: Table S6).

### Measurement of β-carotene production

Production of β-carotene was quantified by measuring absorption of acetone-extracted β-carotene at 453 nm as previously reported [14]. One millililitre of cells cultured for β-carotene production were harvested by centrifugation at 4000 rpm for 10 min, suspended in acetone (1 ml), and incubated at 55 °C for 15 min in dark. Samples were then centrifuged at 14,000 rpm for 10 min to obtain supernatant containing β-carotene, whose absorbance was measured at 453 nm using a Shimadzu UV-2550 spectrophotometer (Shimadzu, Kyoto, Japan). The β-carotene production was normalized to the cell density. The results represented the mean ± SD of three independent experiments.

### Calculation of *mvaS* and *Hmg1* RBS strength of strains from Re-modulation libraries

RBS sequences along with their context region of *mvaS* and *Hmg1* in representative strains were sequenced. Their theoretical RBS strength characterized by the value of translation initiation rate was calculated with the RBS library calculator [29, 30].

## Results

### β-carotene production by *E coli* with heterologous expression of MVA pathway

**Fig. 2:**
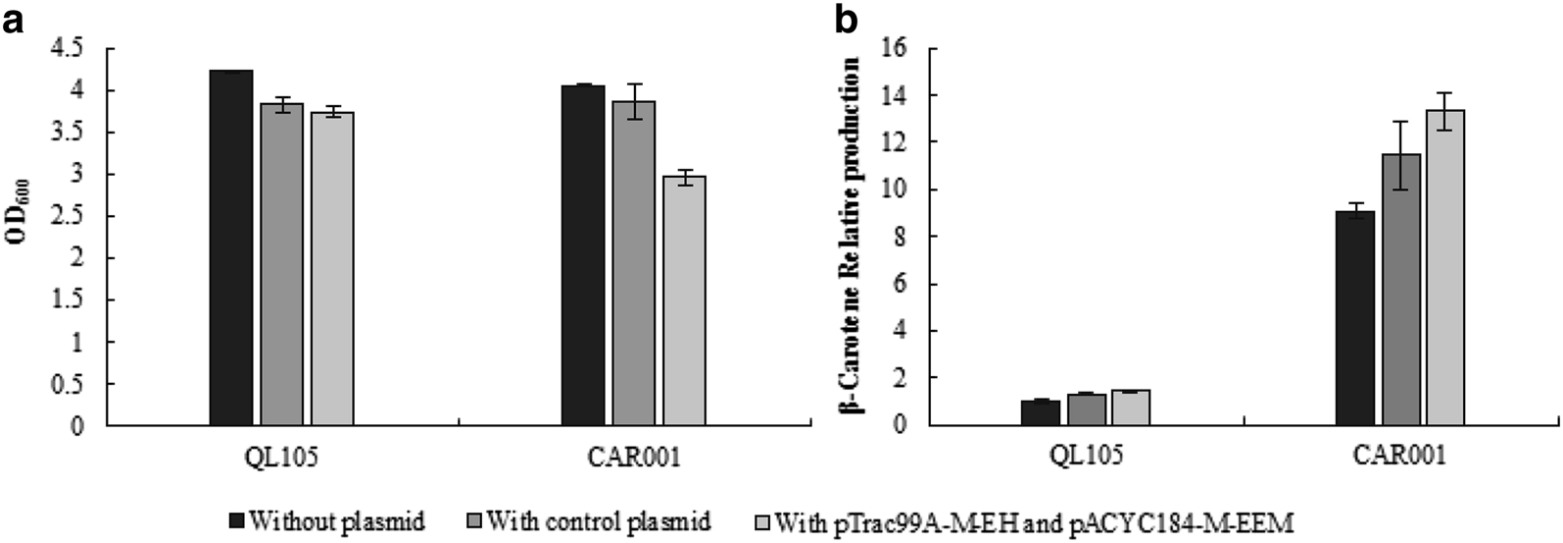
Cell mass and relative β-carotene production by *E. coli* strains with or without MVA pathway. **a** Cell mass. **b** Relative β-carotene production. β-carotene yield was compared to the parent strain QL105 and CAR001. Three repeats were performed for each strain, and the *error bars* represented standard deviation

To eliminate growth inhibition caused by imbalanced overexpression of MVA pathway gene, the heterologous MVA pathway genes from *S. cerevisiae* were separated into two portions and cloned into plasmids pTrac99A-M and pACYC184-M, respectively. pACYC184-M-Mmm containing *mvaS*-*mvaA*-*mavD1* operon and pTrac99A-M-He containing *Hmg1*-*erg12* operon were first transformed into QL105 and CAR001 [14]. Optimization of MEP pathway in QL105 led to increased β-carotene production, and the resulting strain was CAR001 (Fig. 2). Both strains were used as parent strains to compare effects of addition of MVA pathway in present of native and engineered MEP pathway. After culturing with IPTG induction, β-carotene yields of the resulting strain QL105 (pACYC184-M-Mmm, pTrac99A-M-He) and CAR001 (pACYC184-M-Mmm, pTrac99A-M-He) were 1.45-, 1.47-fold of the initial strain (Fig. 2b). Meanwhile, the expression of MVA pathway genes on plasmids caused a 12 and 27% decrease in cell mass for QL105 and CAR001, respectively (Fig. 2a). The result showed that the β-carotene production of the native MEP pathway did not affect introduction of heterologous MVA pathway, in terms of β-carotene production. Growth defect was observed in engineered strains, which were probably due to plasmid burden or imbalanced MVA pathway. In addition, 90% of the plasmid bearing strains lost resistance after 24 h of culturing, indicating loss of their plasmids.

### Improved β-carotene production by Integration MVA pathway genes into *E. coli* and expression modulation

In order to eliminate possible growth burden caused by plasmids and obtain genetically stable strains, the *Hmg1*-*erg12* operon without promoter was integrated into chromosome of CAR001 at *pflB* site, resulting in strain CAR006. Then, this operon was modulated with two regulatory parts (M1-46 and M1-93), resulting in strains CAR007 and CAR008 (Additional file 1: Table S6). Plasmid pACYC184-M-Mmm was transformed into CAR007 and CAR008 to complement MVA pathway. In the resulting strains, the cell mass decreased by nearly 20%, while the β-carotene yield was 1.26- and 1.17-fold that of strain CAR001 respectively (Table 1). To integrate the whole MVA pathway into *E. coli* chromosome, operon of *mvaS*-*mvaA*-*mavD1* genes without promoter was integrated into CAR007 at *frdB* site, resulting in strain CAR009. This operon was then modulated with two regulatory parts (M1-46 and M1-93), resulting strains CAR010 and CAR011 (Additional file 1: Table S6). In the resulting strains, the cell mass decreased by nearly 20%, and the β-carotene yield was only 1.03- and 1.02-fold that of CAR001 respectively (Table 1), suggesting imbalanced expression of the MVA pathway in these two strains.

**Table 1:** Integration and modulation of MVA pathway genes for improving β-carotene production

Strains^a^	OD_600_	OD_453_^b^	OD_453_/OD_600_	Increase of β-carotene yield- CAR001
QL105	4.22 ± 0.15	0.27 ± 0.01	0.06 ± 0.01	
CAR001	4.05 ± 0.10	2.36 ± 0.06	0.58 ± 0.00	1.00 ± 0.00
CAR007 (184-EEM)	3.31 ± 0.05	2.43 ± 0.13	0.73 ± 0.04	1.26 ± 0.06
CAR008 (184-EEM)	3.32 ± 0.07	2.26 ± 0.09	0.68 ± 0.04	1.17 ± 0.07
CAR010	4.05 ± 0.05	2.45 ± 0.07	0.60 ± 0.01	1.03 ± 0.02
CAR011	4.11 ± 0.07	2.43 ± 0.05	0.59 ± 0.02	1.02 ± 0.03
CAR012	4.01 ± 0.07	2.93 ± 0.20	0.73 ± 0.05	1.26 ± 0.09
CAR013	3.91 ± 0.11	2.41 ± 0.12	0.62 ± 0.03	1.07 ± 0.05

^a^Three repeats were performed for each strain, and the error bars represented standard deviation

^b^Acetone-extracted β-carotene solution was concentrated 2 times for measuring the absorption at 453 nm

### Improved β-carotene production by modulation of *atoB* gene expression

*Escherichia coli* is known to contain only low levels of acetoacetyl-CoA [20, 31], which may be the reason for the low β-carotene production of CAR010 and CAR011. To improve β-carotene production, *atoB* of CAR010 was modulated with three regulatory parts (M1-46, M1-37 and M1-93), which were characterized constitutive promoters with different transcription efficiency [26]. The best strain CAR012 with *atoB* expressed by M1-37 had a β-carotene production 1.26-fold that of CAR001 (Table 1).

### Further improvement by re-modulation of MVA pathway genes

Although modulation of all the MVA pathway genes in strain CAR012 with regulatory parts led to an increase in β-carotene production, the increase was only 26%, and the regulatory parts of two operons of the MVA pathway were the same (M1-46). Strengths of these regulatory parts might not be optimal for β-carotene production, suggesting a possibility of obtaining a higher β-carotene production by modulating the expression of these important genes with different artificial regulatory parts.

**Fig. 3:**
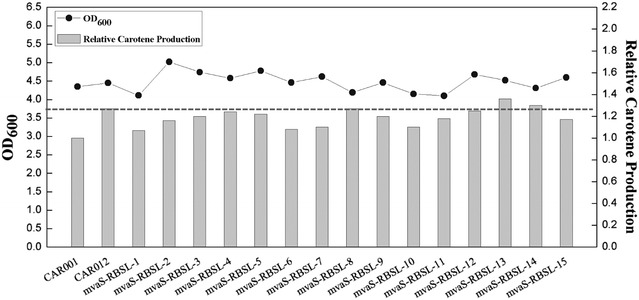
Cell mass and relative β-carotene production by *E. coli* strains after modulating *mvas* expression with RBS library in CAR012

With this strategy, *mvas* gene of the *mvas*-*mvaA*-*mavD1* operon was modulated firstly with an RBS library. Fifteen colonies were randomly selected from the library for measuring β-carotene production (Additional file 1: Table S6), the β-carotene yield ranged from 1.07 to 1.36 times and cell mass ranged from 0.94 to 1.15 times of CAR001 (Fig. 3). The best strain mvaS-RBSL-13, produced 8% higher than CAR012 and 36% higher than CAR001.

**Fig. 4:**
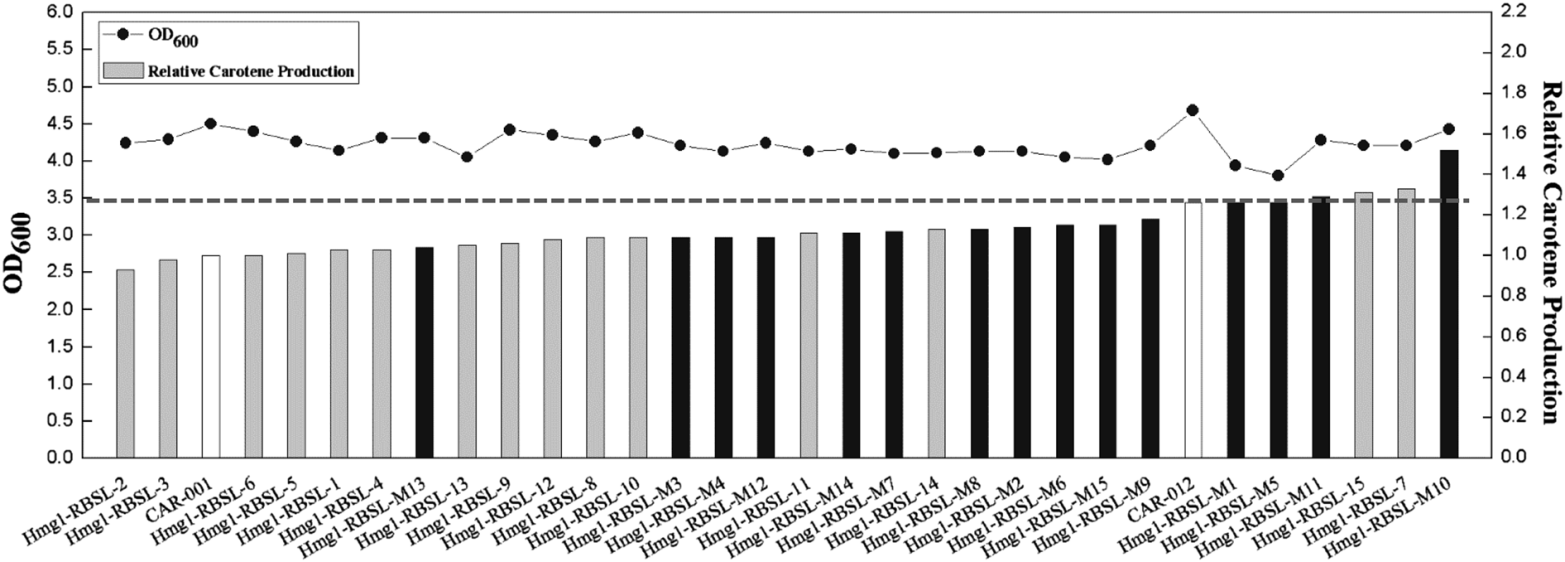
Cell mass and relative production by *E. coli* strains with modulation of *Hmg1* expression with RBS library from mvaS-RBSL-13 or mvaS-RBSL-mix. *Gray Square* recombinant *E. coli* strains derived from mvaS-RBSL-13; *Black square* recombinant *E. coli* strains derived from the mvaS-RBSL-mix pool

To further increase the β-carotene production, *Hmg1* gene *of* the *Hmg1*-*erg12* operon in the best strain mvaS-RBSL-13 was also modulated with the RBS library. In addition, a ten-colony-strain mixture randomly selected from mvaS-RBS Library was used as a parent strain pool for *Hmg1* modulation. Two new libraries were obtained, and fifteen colonies from each library were randomly selected for measuring β-carotene production (Additional file 1: Table S6). The resulting β-carotene yield ranged from 0.93 to 1.51 times and cell mass ranged from 0.90 to 0.98 times of CAR001 (Fig. 4).

**Fig. 5:**
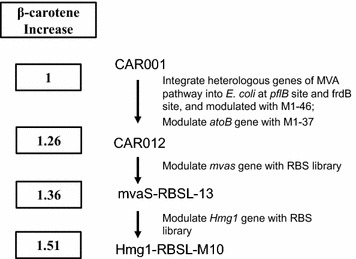
Diagram summarizing steps in the metabolic engineering of *E. coli* for β-carotene production in this work

Gray columns in Fig. 4 represent *E. coli* strains from RBS modulated library of mvaS-RBSL-13, which had β-carotene yield ranged from 0.93 to 1.34 times of CAR001. Most strains had a titer between 0.93 and 1.09 times of CAR001. Black columns represent strains from the RBS modulated library of mvaS-RBSL-mix. Their β-carotene yield ranged from 1.04 to 1.51 times of CAR001, and were mostly between 1.09 and 1.51 times of CAR001. This result suggested that a RBS modulated library from a mixture of strains might be more ideal than those from a single regulatory part, probably due to having more possible regulation patterns. The cell mass of best strains Hmg1-RBSL-M10 was 0.99 times of that of CAR001, which demonstrated that balanced MVA pathway restored growth of the strain (Additional file 1: Table S3). In addition, β-carotene production of best strain Hmg1-RBSL-M10 increased 51% compared with CAR001 (Figs. 4, 5).

Hmg1-RBSL-M10 culture after 48 h fermentation was spread on LB agar plates for measurement of genetic stability. It was found that 100% colonies showing orange color which was the color of β-carotene. This demonstrated the stability of strains with chromosomal integrated heterologous genes. In comparison, when plasmids were used as expression vector we found that 90% strains lost resistance after 24 h of culturing.

### Analysis of *mvaS* and *Hmg1* RBS strength of strains from re-modulation libraries

RBS strength was analyzed with the RBS calculator [29, 30] to find the relationship between *mvaS* and *Hmg1* expression status and β-carotene production. Calculated RBS strength was represented by the translation initiation rate and listed in Table 2 and Additional file 1: Table S7. The RBS strength is by no means a very accurate measurement of the expression status of *mvaS* and *Hmg1*, however, could give a good estimate of general trend of and optimized regulation pattern of MVA pathway. According to the data, a medium to low expression of both *mvaS* and *Hmg1* improved the efficiency of MVA pathway. The best strain carries the third weakest *mvaS* RBS and the middle level of *hmg1* RBS (Additional file 1: Table S7). Since *mvaS* and *Hmg1* are closest to the promoter and had higher transcription than other genes, the weaker RBSs might help to balance their expression with other genes in the same operon, which suggested an overall balanced expression of MVA pathway genes was beneficial with its efficiency.

**Table 2:** Calculated strength of *mvaS* and *Hmg1* RBS from strains from re-modulation libraries

Strains	Sequence of *mvaS* RBS	Strength^a^ of *mvaS* RBS	Sequence of *Hmg1* RBS	Strength^a^ of *Hmg1* RBS	INCREASE of β-carotene yield- CAR001
Hmg1-RBSL-2	AGGAGAGAGAGG	408	AGGAGAAACAAC	3877	0.93
Hmg1-RBSL-3	AGGAGAGAGAGG	408	AGGAGGGAAAAA	9975	0.98
Hmg1-RBSL-6	AGGAGAGAGAGG	408	AGGAGAACAGCT	3238	1.00
Hmg1-RBSL-M3	AGGAGGGTATCG	2159	AGGAGGATAAAG	7965	1.09
Hmg1-RBSL-11	AGGAGAGAGAGG	408	AGGAGGAAAAAC	12,720	1.11
Hmg1-RBSL-M1	AGGAGACAAAAG	1099	AGGAGACAAAAG	2656	1.26
Hmg1-RBSL-15	AGGAGAGAGAGG	408	AGGAGGAAAAGG	4855	1.31
Hmg1-RBSL-M10	AGGAGAGGACTG	612	AGGAGAACAGCT	3238	1.52

^a^ Value of strength was represented by the translation initiation rate calculated by RBS library calculator [29, 30]

## Discussion

In MVA pathway, accumulation of the intermediate 3-hydroxy-3-glutaryl-CoA (HMG-CoA) was found to cause inhibition of cell growth [20]. It is necessary to reduce expression of *mvaS* and increase *Hmg1*, which encodes HMG-CoA reductase, for reduction of HMG-CoA accumulation. The mevalonate should be rapidly converted into mevalonate-5 phosphate by mevalonate kinase (MK) or it would cross cell membrane and diffuse into medium [20]. MK is also one of the rate-limiting enzymes [21]. To facilitate modulation of these genes and balance their expression to keep pathway intermediates at a proper level in host cells, MVA pathway was divided and engineered as three separately expressed operons in this work. This strategy was proved to be effective with increased β-carotene production.

Plasmid overexpression of the MVA pathway genes was previously utilized to increase isoprenoid production in *E. coli*, but caused metabolic burden in host and led to reduced cell growth [32]. In this study, the five heterologous genes of MVA pathway were first cloned into two compatible plasmids, and expressed in QL105 and CAR001 strains. The growth of strains with plasmid had one quarter decrease compared to the initial strains (Fig. 2). In our work, the growth burden was eliminated by integration and of these genes into *E. coli* chromosome followed by modulation. This result indicated that overexpression of heterologous genes with chromosome integrated form was a better strategy than plasmid expression.

Strong promoters have been generally used to overexpress genes for improved carotenoid production [13, 33, 34]. However, they might not be optimal to obtain maximum metabolic flux towards desired products [35]. Previous studies indicated that better production could be obtained by modulating genes with regulatory parts of varied strengths. *dxs* gene of MEP pathway was modulated by several artificial promoters, and an optimal strength for improved lycopene production was identified [36]. Similarly, glyceraldehyde-3-phosphate dehydrogenase gene (*gapA*) was modulated by three artificial promoters to achieve an optimal strength for glycerol production [37]. In both cases, the optimal expression strength was not the strongest one. A similar result was obtained in this work. In the strain with highest β-carotene production, two MVA operons (*mvas*-*mvaD*-*mvaA* and *Hmg1*-*erg12*) were expressed with a medium strength promoters M1-46, and *atoB* was expressed with a weak promoter M1-37. In addition, key enzymes *mvas* and *Hmg1* were under control of medium to weak RBSs. Thus, previous reports and our results suggested an overall balanced expression of MVA pathway genes was relatively efficient, which might also be true for most heterologous pathways.

One of the most important research subjects of metabolic engineering is pursuing a balanced metabolic pathway. In recent years, several combinatorial pathway engineering strategies and methods were established [38, 39]. In this work we dedicated to develop and apply a relatively simple method for pathway balancing and production improvement. Firstly, operons were modulated with limited number of promoters, which were previously defined; then genes within an operon were combinatorially modulated with RBS libraries for multiple rounds. To reduce library size and simplify screening, limited number of strains from previous round were picked as parent strains for next round of modulation with RBS library. By compromising with the goal of finding the most balanced pathways, this method requires minimal lab work for finding a reasonably efficient pathway. In this work, modulating *mvaS* with RBS libraries led to 36% improvement of β-carotene yield versus CAR001, and 8% improvement of β-carotene yield versus parent strain (Figs. 3, 5; Additional file 1: Table S2). This result illustrated that modulating gene with a library of regulatory parts led to a wider variation in expression strength than using limited regulatory parts with fixed strengths, provided more possibilities to obtain optimal efficiency for pathways. Furthermore, a comparison was made between modulating gene expression from a single strain or from a pool of mixed strains in modulating *Hmg1* RBSs. β-carotene production of strains derived from a pool was generally higher than that from a single strain, probably due to similar reasons as above, that more combinations were achieved with parent strains in a mixture pool. This result inspired us to develop methods to build metabolic engineering libraries with more random regulatory parts in higher mathematic dimensions to achieve better possibilities for optimal expression combinations.

In this study, a genetically stable *E. coli* strain with high β-carotene production was obtained by combined engineering of MEP and MVA (Fig. 5). A genetically stable chassis with activated MEP and MVA pathways for IPP and DMAPP precursor supply to produce various terpenes was obtained. Our study provided a novel strategy for improving production of a target compound through integration and subsequent modulation of heterologous pathways by changes in both promoters and RBSs.

## Conclusions

In this study, MVA pathway was divided into three modules, integrated into the *E. coli* chromosome, and individually modulated with regulatory parts to optimize efficiency of the pathway in terms of downstream isoprenoid production. *Hmg1*-*erg12* operon and *mvaS*-*mvaA*-*mavD1* operon were integrated into *E. coli* chromosome followed by modulation with promoters with varied strength, resulting in a 26% increase of β-carotene production with no effect on cell growth. With a combinatory modulation of two key enzymes *mvas* and *Hmg1* with degenerate RBS library, β-carotene showed a further increase of 51%.

Our study provides a novel strategy for improving production of a target compound through integration and modulation of heterologous pathways in both transcription and translation level. In addition, a genetically hard-coded chassis with both efficient MEP and MVA pathways for isoprenoid precursor supply was constructed in this work.

## Additional files

**Additional file 1: Table S1.** Primers used in this work. **Table S2.** Modulating genes of *mvaS*-*mvaA*-*mavD1* operon for improving β-carotene production. **Table S3.** Modulating genes of *Hmg1*-*erg12* operon for improving β-carotene production. **Table S4.** Sequences of representative artificial regulatory parts. **Table S5.** Plasmids used in this work. **Table S6.** *Escherichia coli* strains used in this work. **Table S7.** Calculated strength of *mvaS* and *Hmg1* RBS, RBS sequence and relative β-carotene yield of strains from Re-modulation libraries. **Figure S1.** Two-step recombination method for inserting *Hmg1*-*erg12* operon in *E. coli* chromosome. **Figure S2.** Two-step recombination method for modulating gene expression in *E. coli* chromosome by different artificial regulatory parts.

**Additional file 2.** Additional plasmid profiles and gene sequences.

## Abbreviations

LB: lysogeny broth
IPTG: isopropyl β-d-1-thiogalactopyranoside
RBS: ribosome-binding site
