# Supplementary material for: Combinatory optimization of chromosomal integrated mevalonate pathway for β-carotene production in Escherichia coli
Source: Microb Cell Fact. 2016 Dec 1;15:202. doi: 10.1186/s12934-016-0607-3 (PMC5134235; doi:10.1186/s12934-016-0607-3)
Supplement: Supplementary file 1 — Additional file 1: Table S1. Primers used in this work. Table S2. Modulating genes of mvaS-mvaA-mavD1 operon for improving β-carotene production. Table S3. Modulating genes of Hmg1-erg12 operon for improving β-carotene production. Table S4. Sequences of representative artificial regulatory parts. Table S5. Plasmids used in this work. Table S6. Escherichia coli strains used in this work. Table S7. Calculated strength of mvaS and Hmg1 RBS, RBS sequence and relative β-carotene yield of strains from Re-modulation libraries. Figure S1. Two-step recombination method for inserting Hmg1-erg12 operon in E. coli chromosome. Figure S2. Two-step recombination method for modulating gene expression in E. coli chromosome by different artificial regulatory parts. [file 12934_2016_607_MOESM1_ESM.docx]

**Supplementary information**

**Combinatory Optimization of Chromosomal Integrated Mevalonate Pathway for β-carotene Production in *Escherichia coli***

Lijun Ye^1, 2 a^, Chunzhi Zhang^1 a^, Changhao Bi^2, 3^, Qingyan Li^2, 3*^, Xueli Zhang^2, 3*^

^1^College of Biotechnology, Dalian Polytechnic University, Ministry of Education, Dalian 116034, P R China.

^2^Tianjin Institute of Industrial Biotechnology, Chinese Academy of Sciences, Tianjin 300308, P R China.

^3^ Key Laboratory of Systems Microbial Biotechnology, Chinese Academy of Sciences, Tianjin 300308, P R China.

Table S1 Primers used in this work

| **Primers** | **Sequences** |
| --- | --- |
| **For fusion of the gene of MVA pathway** | |
| ERG13-BamHI-f | CGCGGATCC AGGAGGATTACTAT ATGAAACTCTCAACTAAACTTTGTT |
| ERG13-r | ACTCTGACATTTGATCTGCCTCCTATTTTTTAACATCGTA AGATCTT |
| ERG8-f | TTAAAAAATAGGAGGCAGATCAAATGTCAGAGTTGAGAGCCTT |
| ERG8-r | AACGGTCATGAGTATTACCTCCTATTTATCAAGATAAGTTTCC |
| MVD1-f | TGATAAATAGGAGGTAATACTCATGACCGTTTACACAGCATC |
| MVD1-SalI-r | CGAAGTCGACTATTCCTTTGGTAGACCAGT |
| HMG1-XmaI-f | TCCCCCCGGGAAGGAGATATACCATGGTTTTAACCAATAAAACA |
| HMG1-r | AATGACATGGTATATCTCCTAGGATTTAATGCAGGTGAC |
| ERG12-f | TAAATCCTAGGAGATATACCATGTCATTACCGTTCTTAACT |
| ERG12- SalI-r | ATCGTCGAC TTATGAAGTCCATGGTAAAT |
| ***pflB* gene deletion and integration of *Hmg1-erg12 operon* at *pflB* site** | |
| XZ-pflB-up | TGTCCGAGCTTAATGAAAAGTT |
| XZ-pflB-down | CGAGTAATAACGTCCTGCTGCT |
| XZ-pflB-1 | AAACGGGTAACACCCCAGAC |
| XZ-pflB-2 | CGGAGTGTAAACGTCGAACA |
| 184-cat-up | GCTAGGTACCTGTGACGGAAGATCACTTCG |
| Bs-sacB-down | GCTAGGATCCTTATTTGTTAACTGTTAATTGTC |
| 99A-r | AGAAACGCAAAAAGGCCATC |
| Cat-up | ATGAAACCGCTGATTGCATCTA |
| ***frd* gene deletion and integration of *mvaS-mvaA-mavD1 operon* at *frd* site** | |
| XZ-frdB-up | TGCAGAAAACCATCGACAAG |
| XZ-frdC-down | CACCAATCAG CGTGACAACT |
| XZ-frdC-1 | GCCACCATCGTAATCCTGTT |
| XZ-frdB-2 | ATAGCGCACCACCTCAATTT |
| **For modulation of the gene of MVA pathway** | |
| pflB-up-cat | AAAACGACCACCATTAATGGTTGTCGAAGTACGCAGTAAATAAAAAATCCATGTGACGGAAGATCACTTCGCA |
| pflB-up-P | AAACGACCACCATTAATGGTTGTCGAAGTACGCAGTAAATAAAAAATCCATTATCTCTGGCGGTGTTGAC |
| HMG1 -cat-down | GATAAACTTTTGACTTTCGATCCAGAAATGACTGTTTTATTGGTTAAAACCATTTATTTGTTAACTGTTAATTGTCCT |
| HMG1-RBS-down | GATAAACTTTTGACTTTCGATCCAGAAATGACTGTTTTATTGGTTAAAACCATAGCTGTTTCCTGGTT |
| HMG1-250-r | CCAAAGCGTACAAAGGTAACT |
| Frd-up-P | GAAGGCGAATGGCTGAGATGAAAAACCTGAAAATTGAGGTGGTGCGCTATTTATCTCTGGCGGTGTTGA |
| frdB-cat-up | GAAGGCGAATGGCTGAGATGAAAAACCTGAAAATTGAGGTGGTGCGCTATTGTGACGGAAGATCACTTCGCA |
| MvaS-RBS-down | CTAAGTCTTCCTTTAATACCACACCAACAAAGTTTAGTTGAGAGTTTCATAGCTGTTTCCTGGTT |
| MvaS -cat-down | CTAAGTCTTCCTTTAATACCACACCAACAAAGTTTAGTTGAGAGTTTCATTTATTTGTTAACTGTTAATTGTCCT |
| MvaS -350-r | TTGGTGTCGATGTTGTAACTC |
| **For modulation of *atoB* gene** | |
| atoB-cat-up | TGCTCTTCTCCGGTGTCATTTTCGTCATTGGTTTAACGCTGTTCTGACGGTGTGACGGAAGATCACTTCGCA |
| atoB-cat-down | AAACTACCGATAGCAGTACGTACCGCACTGACGATGACACAATTTTTCATTTATTTGTTAACTGTTAATTGTCCT |
| atoB-p-up | TGCTCTTCTCCGGTGTCATTTTCGTCATTGGTTTAACGCTGTTCTGACGGTTATCTCTGGCGGTGTTGAC |
| atoB-RBS-down | AAACTACCGATAGCAGTACGTACCGCACTGACGATGACACAATTTTTCATAGCTGTTTCCTGGTT |
| AtoB-down | AAACCTGTCCGTCTCCAAGA |
| **For construction artificial regulatory parts with different resistance gene** | |
| FRT-cam-up | AATAGGAACTTCGGAATAGGAACTTCAAGATCCCCTCACGCTGCCGCAAGCATGTGACGGAAGATCACTTC |
| FRT-cam-down | TTCCTATTCTCTAGAAAGTATAGGAACTTCAGAGCGCTTTTGAAGCTGGGGTACTGTGTTAGCGGTCTGC |
| Cat-g-up | TGTGACGGAAGATCACTTC |
| LacZ-373 | AGTAACAACTCGTCGGATTCT |
| FRT-Apra-up | AATAGGAACTTCGGAATAGGAACTTCAAGATCCCCTCACGCTGCCGCAAGCACCGTGATCGAAATCCAGA |
| FRT-Apra-down | TTCCTATTCTCTAGAAAGTATAGGAACTTCAGAGCGCTTTTGAAGCTGGGCGATTACTTCGCCAACTATTG |
| Apra-G-F | CCGTGATCGAAATCCAGA |
| **For modulation of the gene of MVA pathway with RBS library** | |
| pflB-up-FRT | AAACGACCACCATTAATGGTTGTCGAAGTACGCAGTAAATAAAAAATCCAGTGTAGGCTGGAGCTGCTTC |
| HMG1- RBSL-down | GATAAACTTTTGACTTTCGATCCAGAAATGACTGTTTTATTGGTTAAAACCATNNNNNNYCTCCTGGTTTAAACGTACATG |
| frdB-up-FRT | GAAGGCGAATGGCTGAGATGAAAAACCTGAAAATTGAGGTGGTGCGCTATGTGTAGGCTGGAGCTGCTTC |
| Mvas-RBSL-down | CTAAGTCTTCCTTTAATACCACACCAACAAAGTTTAGTTGAGAGTTTCATNNNNNNYCTCCTGGTTTAAACGTACATG |

Table S2 Modulating genes of *mvaS-mvaA-mavD1 operon* for improving β-carotene production.

| Strains^a^ | OD_600_ | OD_453_^b^ | OD_453_/OD_600_ | Increase of β-carotene yield- CAR012 | Increase of β-carotene yield- CAR001 |
| --- | --- | --- | --- | --- | --- |
| CAR001 | 4.35 | 2.12 | 0.49 | 0.79 | 1.00 |
| CAR012 | 4.45 | 2.50 | 0.56 | 1.00 | 1.27 |
| mvaS-RBSL-1 | 4.11 | 2.14 | 0.52 | 0.84 | 1.07 |
| mvaS-RBSL-2 | 5.02 | 2.84 | 0.57 | 0.91 | 1.16 |
| mvaS-RBSL-3 | 4.74 | 2.77 | 0.58 | 0.94 | 1.20 |
| mvaS-RBSL-4 | 4.58 | 2.76 | 0.60 | 0.97 | 1.24 |
| mvaS-RBSL-5 | 4.78 | 2.83 | 0.59 | 0.96 | 1.22 |
| mvaS-RBSL-6 | 4.46 | 2.34 | 0.52 | 0.85 | 1.08 |
| mvaS-RBSL-7 | 4.62 | 2.47 | 0.53 | 0.86 | 1.10 |
| mvaS-RBSL-8 | 4.19 | 2.58 | 0.62 | 1.00 | 1.27 |
| mvaS-RBSL-9 | 4.46 | 2.61 | 0.59 | 0.95 | 1.20 |
| mvaS-RBSL-10 | 4.15 | 2.22 | 0.53 | 0.87 | 1.10 |
| mvaS-RBSL-11 | 4.10 | 2.36 | 0.58 | 0.93 | 1.18 |
| mvaS-RBSL-12 | 4.68 | 2.85 | 0.61 | 0.99 | 1.25 |
| mvaS-RBSL-13 | 4.52 | 2.92 | 0.65 | 1.07 | 1.36 |
| mvaS-RBSL-14 | 4.31 | 2.73 | 0.63 | 1.03 | 1.30 |
| mvaS-RBSL-15 | 4.60 | 2.62 | 0.57 | 0.92 | 1.17 |

^a^ fifteen strains were selected from *mvaS* RBS library at random, named from mvaS-RBSL-1 to mvaS-RBSL-15.

^b^ Acetone-extracted β-carotene solution was concentrated 2 times for measuring the absorption at 453 nm.

Table S3 Modulating genes of *Hmg1-erg12* operon for improving β-carotene production.

| Strains | OD_600_ | OD_453_^c^ | OD_453_/OD_600_ | Increase of β-carotene yield- CAR012 | Increase of β-carotene yield- CAR001 |
| --- | --- | --- | --- | --- | --- |
| CAR-001 | 4.50 | 2.14 | 0.48 | 0.79 | 1 |
| CAR-012 | 4.68 | 2.82 | 0.60 | 1 | 1.26 |
| mvaS-RBSL-13 | 4.52 | 2.92 | 0.65 | 1.07 | 1.36 |
| **Modulating the *Hmg1-erg12 operon* with RBS in mvaS-RBSL-13^a^** | | | | | |
| Hmg1-RBSL-1 | 4.14 | 2.04 | 0.49 | 0.82 | 1.03 |
| Hmg1-RBSL-2 | 4.24 | 1.88 | 0.44 | 0.73 | 0.93 |
| Hmg1-RBSL-3 | 4.29 | 2.01 | 0.47 | 0.78 | 0.98 |
| Hmg1-RBSL-4 | 4.31 | 2.12 | 0.49 | 0.81 | 1.03 |
| Hmg1-RBSL-5 | 4.26 | 2.04 | 0.48 | 0.80 | 1.01 |
| Hmg1-RBSL-6 | 4.40 | 2.10 | 0.48 | 0.79 | 1.00 |
| Hmg1-RBSL-7 | 4.21 | 2.68 | 0.64 | 1.06 | 1.33 |
| Hmg1-RBSL-8 | 4.26 | 2.20 | 0.52 | 0.86 | 1.09 |
| Hmg1-RBSL-9 | 4.42 | 2.24 | 0.51 | 0.84 | 1.06 |
| Hmg1-RBSL-10 | 4.38 | 2.28 | 0.52 | 0.86 | 1.09 |
| Hmg1-RBSL-11 | 4.13 | 2.18 | 0.53 | 0.88 | 1.11 |
| Hmg1-RBSL-12 | 4.35 | 2.24 | 0.51 | 0.86 | 1.08 |
| Hmg1-RBSL-13 | 4.05 | 2.02 | 0.50 | 0.83 | 1.05 |
| Hmg1-RBSL-14 | 4.11 | 2.21 | 0.54 | 0.89 | 1.13 |
| Hmg1-RBSL-15 | 4.21 | 2.63 | 0.62 | 1.04 | 1.31 |
| **Modulating the *Hmg1-erg12 operon* with RBS in mvaS-RBSL-mix^b^** | | | | | |
| Hmg1-RBSL-M1 | 3.94 | 2.36 | 0.60 | 0.99 | 1.26 |
| Hmg1-RBSL-M2 | 4.13 | 2.24 | 0.54 | 0.90 | 1.14 |
| Hmg1-RBSL-M3 | 4.21 | 2.85 | 0.68 | 0.87 | 1.09 |
| Hmg1-RBSL-M4 | 4.13 | 2.15 | 0.52 | 0.87 | 1.09 |
| Hmg1-RBSL-M5 | 3.80 | 2.28 | 0.60 | 1.00 | 1.26 |
| Hmg1-RBSL-M6 | 4.05 | 2.22 | 0.55 | 0.91 | 1.15 |
| Hmg1-RBSL-M7 | 4.10 | 2.18 | 0.53 | 0.88 | 1.12 |
| Hmg1-RBSL-M8 | 4.13 | 2.23 | 0.54 | 0.90 | 1.13 |
| Hmg1-RBSL-M9 | 4.21 | 2.37 | 0.56 | 0.94 | 1.18 |
| Hmg1-RBSL-M10 | 4.43 | 3.20 | 0.72 | 1.20 | 1.52 |
| Hmg1-RBSL-M11 | 4.28 | 2.62 | 0.61 | 1.02 | 1.29 |
| Hmg1-RBSL-M12 | 4.24 | 2.20 | 0.52 | 0.86 | 1.09 |
| Hmg1-RBSL-M13 | 4.31 | 2.13 | 0.49 | 0.82 | 1.04 |
| Hmg1-RBSL-M14 | 4.16 | 2.19 | 0.53 | 0.87 | 1.11 |
| Hmg1-RBSL-M15 | 4.02 | 2.20 | 0.55 | 0.91 | 1.15 |

^a^ the *hmg1* gene of the mvaS-RBSL-13 was modulated by RBS library, then fifteen strains were selected from the library at random, named from Hmg1-RBSL-1 to Hmg1-RBSL-15.

^b^ ten colonies were chose from *mvaS* RBS library at random, then the *hmg1* gene of the mix strains was modulated with RBS library, and fifteen strains were selected from the library at random, named from Hmg1-RBSL-M1 to Hmg1-RBSL-M15.

^c^ Acetone-extracted β-carotene solution was concentrated 2 times for measuring the absorption at 453 nm.

Table S4 Sequences of representative artificial regulatory parts.

| **Regulatory parts** | **Sequences** |
| --- | --- |
|  | **Promoter^a^** |
| M1-37 | TTATCTCTGGCGGTG**TTGACA**AGAGATAACAACGTTGA**TATAAT**  TGAGCCACTGGCTCGTAATTTATTGTTTAAAC*CAGGAAACAGCT* |
| M1-46 | TTATCTCTGGCGGTG**TTGACA**AGAGATAACAACGTTGA**TATAAT**  TGAGCCTCTCGCCCCACCAATTCGGTTTAAAC*CAGGAAACAGCT* |
| M1-93 | TTATCTCTGGCGGTG**TTGACA**AGAGATAACAACGTTGA**TATAAT**  TGAGCCCGTATTGTTAGCATGTACGTTTAAAC*CAGGAAACAGCT* |
|  | **RBS^b^** |
| RBSL | AGGAG**RNNNNNN**+ATG |

^a^ The −35 and −10 consensus sequences are in bold, the mRS sequences are underlined, and the ribosomal binding site sequences are italicized.

^b^ The RBS sequence is underlined, and the random sequence is bold. The “R” represents bases “A/G”, and the “N” represents bases “A/G/C/T”.

Table S5 Plasmids used in this work.

| **Plasmids** | **Relative characteristics** | **Sources** |
| --- | --- | --- |
| pKD46 | bla*γ β exo* (Red recombinase), temperature-conditional replicon | [Datsenko and Wanner et al. (2000](#_ENREF_9)) |
| pKD4 | *bla*; FRT-*Kan*-FRT | [Datsenko and Wanner et al. (2000](#_ENREF_9)) |
| pLOI4162 | *bla; cat-sacB* cassette | Jantama et al. (2008) |
| pTrc99A-M | *bla*; high-copy expression plasmid | Zhao et al. (2013) |
| pACYC184-M | *cat*; low-copy expression plasmid | Zhao et al. (2013) |
| pTrc99A-M-He | *bla; Hmg1* and *erg12* amplified from *Saccharomyces cerevisiae* cloned into pTrc99A-M at *Xma*I and *Sal*I site | This work |
| pACYC184-M-Mmm | *cat*; *mvas, mvaA* and *mavD* amplified from *Saccharomyces cerevisiae* cloned into pACYC184-M at *Bam*HI and *Sal*I site | This work |
| pXZ014 | Bla: kan; *pflB* (PCR) from *E. coli* ATCC8739 (XZ-pflB-up/XZ-pflB-down) cloned into pEASY-Blunt vector | Shi et al. (2013) |
| pXZ015C | *cat-sacB* cassette (cat-sacB-up/cat-sacB-down) from pXZ-CS cloned into *pflB* fragment of pXZ014 (XZ-pflB-1/XZ-pflB-2) | Tan et al. |
| pQL003-He | *Hmg1-erg12 operon* from pTrc99A-M-He cloned into *pflB* fragment of pXZ014 (XZ-pflB-1/XZ-pflB-2) | This work |
| pXZ005 | *Bla*: *kan*; *frdABCD*(XZ-frdB-up/XZ-frdC-down) from *E. coli* ATCC8739 cloned into pEASY-Blunt vector | Shi et al. (2013) |
| pXZ006C | *cat-sacB* cassette (184-cat-up /Bs-sacB-down) from pXZ-CS cloned into *frdBC* (XZ-frdC-1/XZ-frdB-2) of pXZ005 | This work |
| pQL006-Mmm | *mvaS-mvaA-mavD1 operon* from pACYC184-M-Mmm cloned into *frdBC (XZ-frdC-1/XZ-frdB-2)* of pXZ005 | This work |

Table S6 *Escherichia coli* strains used in this work

| **strains** | **Relative characteristics** | **Sources** |
| --- | --- | --- |
| ATCC 8739 | Wild type | Jantama et al., 2008 |
| M1-12 | ATCC 8739, FRT-Km-FRT::M1-12::*lacZ* | Lu et al., 2012 |
| M1-46 | ATCC 8739, FRT-Km-FRT::M1-46::*lacZ* | Lu et al., 2012 |
| M1-37 | ATCC 8739, FRT-Km-FRT::M1-37::*lacZ* | Lu et al., 2012 |
| M1-93 | ATCC 8739, FRT-Km-FRT::M1-93::*lacZ* | Lu et al., 2012 |
| QL105 | ATCC 8739, *ldhA::*M1-12*::crtEXYIB::ldhA* | Zhao et al., 2013 |
| Dxs37-Idi46 | QL105, M1-37::*dxs*, M1-46::*idi* | Zhao et al., 2013 |
| CAR001 | Dxs37-Idi46, M1-93::*crtEXYIB* | Zhao et al., 2013 |
| M1-cam-93 | ATCC 8739, FRT-Cam-FRT::M1-93::*lacZ* | This work |
| M1-Apr-93 | ATCC 8739, FRT-apr-FRT::M1-93::*lacZ* | This work |
| **Engineering β-carotene synthesis and MEP modules** | | |
| CAR006 | CAR001, *pflB::Hmg1,erg12* | This work |
| CAR007 | CAR001, *pflB::M1-46-Hmg1,erg12* | This work |
| CAR008 | CAR001, *pflB::M1-93-Hmg1,erg12* | This work |
| CAR009 | CAR007, *frdB::mvas,mvaD,mvaA* | This work |
| CAR010 | CAR007, *frdB:: M1-46-mvas,mvaD,mvaA* | This work |
| CAR011 | CAR007, *frdB:: M1-93-mvas,mvaD,mvaA* | This work |
| CAR012 | CAR010, *M1-37-atoB* | This work |
| CAR013 | CAR010, *M1-46-atoB* | This work |
| CAR014 | CAR010, *M1-93-atoB* | This work |
| **Modulating the *mvaS-mvaA-mavD1 operon* by RBS library inCAR012** | | |
| mvaS-RBSL-1 | CAR012, *frdB::M1-RBS-1-mvas, mvaD, mvaA* | This work |
| mvaS-RBSL-2 | CAR012, *frdB::M1-RBS-2-mvas,mvaD,mvaA* | This work |
| mvaS-RBSL-3 | CAR012, *frdB::M1-RBS-3-mvas, mvaD, mvaA* | This work |
| mvaS-RBSL-4 | CAR012, *frdB::M1-RBS-4-mvas, mvaD, mvaA* | This work |
| mvaS-RBSL-5 | CAR012, *frdB::M1-RBS-5-mvas, mvaD, mvaA* | This work |
| mvaS-RBSL-6 | CAR012, *frdB::M1-RBS-6-mvas, mvaD, mvaA* | This work |
| mvaS-RBSL-7 | CAR012, *frdB::M1-RBS-7-mvas, mvaD, mvaA* | This work |
| mvaS-RBSL-8 | CAR012, *frdB::M1-RBS-8-mvas, mvaD, mvaA* | This work |
| mvaS-RBSL-9 | CAR012, *frdB::M1-RBS-9-mvas, mvaD, mvaA* | This work |
| mvaS-RBSL-10 | CAR012, *frdB::M1-RBS-10-mvas, mvaD, mvaA* | This work |
| mvaS-RBSL-11 | CAR012, *frdB::M1-RBS-11-mvas, mvaD, mvaA* | This work |
| mvaS-RBSL-12 | CAR012, *frdB::M1-RBS-12-mvas, mvaD, mvaA* | This work |
| mvaS-RBSL-13 | CAR012, *frdB::M1-RBS-13-mvas, mvaD, mvaA* | This work |
| mvaS-RBSL-14 | CAR012, *frdB::M1-RBS-14-mvas, mvaD, mvaA* | This work |
| mvaS-RBSL-15 | CAR012, *frdB::M1-RBS-15-mvas, mvaD, mvaA* | This work |
| **Modulating the *Hmg1-erg12 operon* by RBS in mvaS-RBSL-13** | | |
| Hmg1-RBSL-1 | mvaS-RBSL-13, *pflB::M1-RBS-1-Hmg1, erg12* | This work |
| Hmg1-RBSL-2 | mvaS-RBSL-13, *pflB::M1-RBS-2-Hmg1, erg12* | This work |
| Hmg1-RBSL-3 | mvaS-RBSL-13, *pflB::M1-RBS-3-Hmg1, erg12* | This work |
| Hmg1-RBSL-4 | mvaS-RBSL-13, *pflB::M1-RBS-4-Hmg1, erg12* | This work |
| Hmg1-RBSL-5 | mvaS-RBSL-13, *pflB::M1-RBS-5-Hmg1, erg12* | This work |
| Hmg1-RBSL-6 | mvaS-RBSL-13, *pflB::M1-RBS-6-Hmg1, erg12* | This work |
| Hmg1-RBSL-7 | mvaS-RBSL-13, *pflB::M1-RBS-7-Hmg1, erg12* | This work |
| Hmg1-RBSL-8 | mvaS-RBSL-13, *pflB::M1-RBS-8-Hmg1, erg12* | This work |
| Hmg1-RBSL-9 | mvaS-RBSL-13, *pflB::M1-RBS-9-Hmg1, erg12* | This work |
| Hmg1-RBSL-10 | mvaS-RBSL-13, *pflB::M1-RBS-10-Hmg1, erg12* | This work |
| Hmg1-RBSL-11 | mvaS-RBSL-13, *pflB::M1-RBS-11-Hmg1, erg12* | This work |
| Hmg1-RBSL-12 | mvaS-RBSL-13, *pflB::M1-RBS-12-Hmg1, erg12* | This work |
| Hmg1-RBSL-13 | mvaS-RBSL-13, *pflB::M1-RBS-13-Hmg1, erg12* | This work |
| Hmg1-RBSL-14 | mvaS-RBSL-13, *pflB::M1-RBS-14-Hmg1, erg12* | This work |
| Hmg1-RBSL-15 | mvaS-RBSL-13, *pflB::M1-RBS-15-Hmg1, erg12* | This work |
| **Modulating the *Hmg1-erg12 operon* by RBS library in mvaS-RBSL-mix** | | |
| Hmg1-RBSL-M1 | mvaS-RBSL-mix, *pflB::M1-RBS-1-Hmg1, erg12* | This work |
| Hmg1-RBSL-M2 | mvaS-RBSL-mix, *pflB::M1-RBS-2-Hmg1, erg12* | This work |
| Hmg1-RBSL-M3 | mvaS-RBSL-mix, *pflB::M1-RBS-3-Hmg1, erg12* | This work |
| Hmg1-RBSL-M4 | mvaS-RBSL-mix, *pflB::M1-RBS-4-Hmg1, erg12* | This work |
| Hmg1-RBSL-M5 | mvaS-RBSL-mix, *pflB::M1-RBS-5-Hmg1, erg12* | This work |
| Hmg1-RBSL-M6 | mvaS-RBSL-mix, *pflB::M1-RBS-6-Hmg1, erg12* | This work |
| Hmg1-RBSL-M7 | mvaS-RBSL-mix, *pflB::M1-RBS-7-Hmg1, erg12* | This work |
| Hmg1-RBSL-M8 | mvaS-RBSL-mix, *pflB::M1-RBS-8-Hmg1, erg12* | This work |
| Hmg1-RBSL-M9 | mvaS-RBSL-mix, *pflB::M1-RBS-9-Hmg1, erg12* | This work |
| Hmg1-RBSL-M10 | mvaS-RBSL-mix, *pflB::M1-RBS-10-Hmg1, erg12* | This work |
| Hmg1-RBSL-M11 | mvaS-RBSL-mix, *pflB::M1-RBS-11-Hmg1, erg12* | This work |
| Hmg1-RBSL-M12 | mvaS-RBSL-mix, *pflB::M1-RBS-12-Hmg1, erg12* | This work |
| Hmg1-RBSL-M13 | mvaS-RBSL-mix, *pflB::M1-RBS-13-Hmg1, erg12* | This work |
| Hmg1-RBSL-M14 | mvaS-RBSL-mix, *pflB::M1-RBS-14-Hmg1, erg12* | This work |
| Hmg1-RBSL-M15 | mvaS-RBSL-mix, *pflB::M1-RBS-15-Hmg1, erg12* | This work |

Table S7. Calculated strength of *mvaS* and *Hmg1* RBS, RBS sequence and relative β-carotene yield of strains from Re-modulation libraries

| Strains | sequence of *mvaS* RBS | strength of mvaS RBS^a^ | sequence of *Hmg1* RBS | strength of Hmg1 RBS^a^ | Increase of β-carotene yield- CAR001 |
| --- | --- | --- | --- | --- | --- |
| Hmg1-RBSL-2 | AGGAGAGAGAGG | 408 | AGGAGAAACAAC | 3877 | 0.93 |
| Hmg1-RBSL-3 | AGGAGAGAGAGG | 408 | AGGAGGGAAAAA | 9975 | 0.98 |
| Hmg1-RBSL-6 | AGGAGAGAGAGG | 408 | AGGAGAACAGCT | 3238 | 1.00 |
| Hmg1-RBSL-5 | AGGAGAGAGAGG | 408 | AGGAGACTAAAC | 625 | 1.01 |
| Hmg1-RBSL-1 | AGGAGAGAGAGG | 408 | AGGAGGAAAAAA | 6044 | 1.03 |
| Hmg1-RBSL-4 | AGGAGAGAGAGG | 408 | AGGAAACAGCT | 397 | 1.03 |
| Hmg1-RBSL-M13 | AGGAGAGACGGA | 1973 | AGGAGGGGAAGA | 3785 | 1.04 |
| Hmg1-RBSL-13 | AGGAGAGAGAGG | 408 | AGGAAACAGCT | 397 | 1.05 |
| Hmg1-RBSL-9 | AGGAGAGAGAGG | 408 | AGGAAACAGCT | 397 | 1.06 |
| Hmg1-RBSL-12 | AGGAGAGAGAGG | 408 | AGGAGAAACGAA | 1344 | 1.08 |
| Hmg1-RBSL-8 | AGGAGAGAGAGG | 408 | AGGAGAATGACA | 243 | 1.09 |
| Hmg1-RBSL-10 | AGGAGAGAGAGG | 408 | AGGAGGGTAGAC | 1761 | 1.09 |
| Hmg1-RBSL-M3 | AGGAGGGTATCG | 2159 | AGGAGGATAAAG | 7965 | 1.09 |
| Hmg1-RBSL-M4 | AGGAGAACGCCA | 608 | AGGAGGAAAAGA | 4531 | 1.09 |
| Hmg1-RBSL-M12 | AGGAGAACGCCA | 608 | AGGAGGAAAATA | 5675 | 1.09 |
| Hmg1-RBSL-11 | AGGAGAGAGAGG | 408 | AGGAGGAAAAAC | 12720 | 1.11 |
| Hmg1-RBSL-M14 | AGGAGAACGCCA | 608 | AGGAGGACGAAA | 1524 | 1.11 |
| Hmg1-RBSL-M7 | AGGAGAACGCCA | 608 | AGGAGGAAAAAG | 3785 | 1.12 |
| Hmg1-RBSL-14 | AGGAGAGAGAGG | 408 | AGGAGAAACAAC | 2205 | 1.13 |
| Hmg1-RBSL-M8 | AGGAGAACGCCA | 608 | AGGAGAAGAAAG | 812 | 1.13 |
| Hmg1-RBSL-M2 | AGGAGAGACGGA | 1973 | AGGAGGAAAAAG | 3785 | 1.14 |
| Hmg1-RBSL-M6 | AGGAGAACGCCA | 608 | AGGAGGAACAAA | 5675 | 1.15 |
| Hmg1-RBSL-M15 | AGGAGAACGCCA | 608 | AGGAGGAGGAAA | 2889 | 1.15 |
| Hmg1-RBSL-M9 | AGGAGAACGCCA | 608 | AGGAGGAACGGC | 2307 | 1.18 |
| Hmg1-RBSL-M1 | AGGAGACAAAAG | 1099 | AGGAGACAAAAG | 2656 | 1.26 |
| Hmg1-RBSL-M5 | AGGAGAACGCCA | 608 | AGGAGGAAAAGA | 4531 | 1.26 |
| Hmg1-RBSL-M11 | AGGAGAGACGGA | 1973 | AGGAGGACATTG | 1471 | 1.29 |
| Hmg1-RBSL-15 | AGGAGAGAGAGG | 408 | AGGAGGAAAAGG | 4855 | 1.31 |
| Hmg1-RBSL-7 | AGGAGAGAGAGG | 408 | AGGAGGAAAAGG | 2307 | 1.33 |
| Hmg1-RBSL-M10 | AGGAGAGGACTG | 612 | AGGAGAACAGCT | 3238 | 1.52 |

^a^ Value of Strength was represented by the Translation Initiation Rate calculated by RBS Library Calculator [29, 30].

Fig. S1

pflB-up

pXZ015C

*Cat-sacB*

**B**

*Recombinant E. coli*

chromosome

pQL003-He

Gene-cat-sacB

Gene-cat-sacB

**A**

pflB-cat-sacB

pflB-up

pflB-up

pflB-down

*Cat-sacB*

pflB-up

pflB-up

*pflB*

pflB-up

pflB-up

*Cat-sacB*

pflB-up

pflB-up

pflB-up

*Hmg1 erg12*

pflB-up

pflB-up

pflB-down

*Cat-sacB*

pflB-up

pflB-up

*Hmg1 erg12*

pflB-up

pflB-up

*Hmg1 erg12*

pflB-up

pflB-up

CAR006

**Fig S1.** Two-step recombination method for inserting *Hmg1* and *erg12* gene operon in *E. coli* chromosome. A, The first recombination step, *cat-sacB* cassette was amplified and used to replace *pflB* gene in CAR001. B, The second recombination step, the *Hmg1* and *erg12* gene together with the outside sequence of *pflB* gene were amplified and used to replace *cat-sacB* cassette.

Fig S2

pflB-up-cat

pXZ-CS

Hmg1-cat-down

pflB-up

Hmg1

*Cat-sacB*

Hmg1

pflB-up

*Cat-sacB*

**B**

CAR006

*Km*

FRT

FRT

RBS

*lacZ’*

M-Lib1

pflB-up-P

Gene-cat-sacB

RBS

Hmg1

Gene-cat-sacB

**A**

Gene-cat-sacB

Aritficial regulatory part

Aritficial regulatory part

Hmg1-RBS-down

pflB-up

Hmg1

*Cat-sacB*

pflB-up

Hmg1

*Cat-sacB*

pflB-up

RBS

Hmg1

Aritficial regulatory part

pflB-up

**Fig S2.** Two-step recombination method for modulating gene expression in *E. coli* chromosome by different artificial regulatory parts. A, The first recombination step, *cat-sacB* cassette was amplified and used to inserted in front of *Hmg1* in CAR006. B, The second recombination step, different artificial regulatory parts were amplified and used to replace *cat-sacB* cassette.
