# Supplementary material for: Combinatory optimization of chromosomal integrated mevalonate pathway for β-carotene production in Escherichia coli
Source: Microb Cell Fact. 2016 Dec 1;15:202. doi: 10.1186/s12934-016-0607-3 (PMC5134235; doi:10.1186/s12934-016-0607-3)
Supplement: Supplementary file 2 — Additional file 2. Additional plasmid profiles and gene sequences. [file 12934_2016_607_MOESM2_ESM.docx]

**Additional plasmid profiles and gene sequences**

pTrc99A-M-He


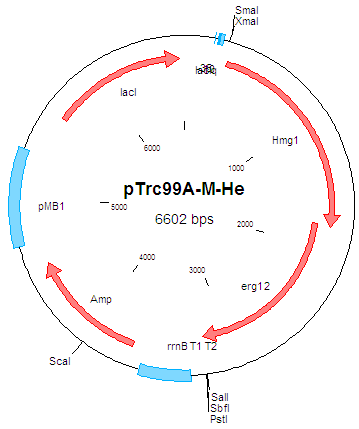


>pTrc99A-M-He

GTTTGACAGCTTATCATCGACTGCACGGTGCACCAATGCTTCTGGCGTCA

GGCAGCCATCGGAAGCTGTGGTATGGCTGTGCAGGTCGTAAATCACTGCA

TAATTCGTGTCGCTCAAGGCGCACTCCCGTTCTGGATAATGTTTTTTGCG

CCGACATCATAACGGTTCTGGCAAATATTCTGAAATGAGCTGTTGACAAT

TAATCATCCGGCTCGTATAATGTGTGGAATTGTGAGCGGATAACAATTTC

ACACAGGAAACAGACCATGGAATTCGAGCTCGGTACCCGGGAAGGAGATA

TACCATGGTTTTAACCAATAAAACAGTCATTTCTGGATCGAAAGTCAAAA

GTTTATCATCTGCGCAATCGAGCTCATCAGGACCTTCATCATCTAGTGAG

GAAGATGATTCCCGCGATATTGAAAGCTTGGATAAGAAAATACGTCCTTT

AGAAGAATTAGAAGCATTATTAAGTAGTGGAAATACAAAACAATTGAAGA

ACAAAGAGGTCGCTGCCTTGGTTATTCACGGTAAGTTACCTTTGTACGCT

TTGGAGAAAAAATTAGGTGATACTACGAGAGCGGTTGCGGTACGTAGGAA

GGCTCTTTCAATTTTGGCAGAAGCTCCTGTATTAGCATCTGATCGTTTAC

CATATAAAAATTATGACTACGACCGCGTATTTGGCGCTTGTTGTGAAAAT

GTTATAGGTTACATGCCTTTGCCCGTTGGTGTTATAGGCCCCTTGGTTAT

CGATGGTACATCTTATCATATACCAATGGCAACTACAGAGGGTTGTTTGG

TAGCTTCTGCCATGCGTGGCTGTAAGGCAATCAATGCTGGCGGTGGTGCA

ACAACTGTTTTAACTAAGGATGGTATGACAAGAGGCCCAGTAGTCCGTTT

CCCAACTTTGAAAAGATCTGGTGCCTGTAAGATATGGTTAGACTCAGAAG

AGGGACAAAACGCAATTAAAAAAGCTTTTAACTCTACATCAAGATTTGCA

CGTCTGCAACATATTCAAACTTGTCTAGCAGGAGATTTACTCTTCATGAG

ATTTAGAACAACTACTGGTGACGCAATGGGTATGAATATGATTTCTAAAG

GTGTCGAATACTCATTAAAGCAAATGGTAGAAGAGTATGGCTGGGAAGAT

ATGGAGGTTGTCTCCGTTTCTGGTAACTACTGTACCGACAAAAAACCAGC

TGCCATCAACTGGATCGAAGGTCGTGGTAAGAGTGTCGTCGCAGAAGCTA

CTATTCCTGGTGATGTTGTCAGAAAAGTGTTAAAAAGTGATGTTTCCGCA

TTGGTTGAGTTGAACATTGCTAAGAATTTGGTTGGATCTGCAATGGCTGG

GTCTGTTGGTGGATTTAACGCACATGCAGCTAATTTAGTGACAGCTGTTT

TCTTGGCATTAGGACAAGATCCTGCACAAAATGTTGAAAGTTCCAACTGT

ATAACATTGATGAAAGAAGTGGACGGTGATTTGAGAATTTCCGTATCCAT

GCCATCCATCGAAGTAGGTACCATCGGTGGTGGTACTGTTCTAGAACCAC

AAGGTGCCATGTTGGACTTATTAGGTGTAAGAGGCCCGCATGCTACCGCT

CCTGGTACCAACGCACGTCAATTAGCAAGAATAGTTGCCTGTGCCGTCTT

GGCAGGTGAATTATCCTTATGTGCTGCCCTAGCAGCCGGCCATTTGGTTC

AAAGTCATATGACCCACAACAGGAAACCTGCTGAACCAACAAAACCTAAC

AATTTGGACGCCACTGATATAAATCGTTTGAAAGATGGGTCCGTCACCTG

CATTAAATCCTAGGAGATATACCATGTCATTACCGTTCTTAACTTCTGCA

CCGGGAAAGGTTATTATTTTTGGTGAACACTCTGCTGTGTACAACAAGCC

TGCCGTCGCTGCTAGTGTGTCTGCGTTGAGAACCTACCTGCTAATAAGCG

AGTCATCTGCACCAGATACTATTGAATTGGACTTCCCGGACATTAGCTTT

AATCATAAGTGGTCCATCAATGATTTCAATGCCATCACCGAGGATCAAGT

AAACTCCCAAAAATTGGCCAAGGCTCAACAAGCCACCGATGGCTTGTCTC

AGGAACTCGTTAGTCTTTTGGATCCGTTGTTAGCTCAACTATCCGAATCC

TTCCACTACCATGCAGCGTTTTGTTTCCTGTATATGTTTGTTTGCCTATG

CCCCCATGCCAAGAATATTAAGTTTTCTTTAAAGTCTACTTTACCCATCG

GTGCTGGGTTGGGCTCAAGCGCCTCTATTTCTGTATCACTGGCCTTAGCT

ATGGCCTACTTGGGGGGGTTAATAGGATCTAATGACTTGGAAAAGCTGTC

AGAAAACGATAAGCATATAGTGAATCAATGGGCCTTCATAGGTGAAAAGT

GTATTCACGGTACCCCTTCAGGAATAGATAACGCTGTGGCCACTTATGGT

AATGCCCTGCTATTTGAAAAAGACTCACATAATGGAACAATAAACACAAA

CAATTTTAAGTTCTTAGATGATTTCCCAGCCATTCCAATGATCCTAACCT

ATACTAGAATTCCAAGGTCTACAAAAGATCTTGTTGCTCGCGTTCGTGTG

TTGGTCACCGAGAAATTTCCTGAAGTTATGAAGCCAATTCTAGATGCCAT

GGGTGAATGTGCCCTACAAGGCTTAGAGATCATGACTAAGTTAAGTAAAT

GTAAAGGCACCGATGACGAGGCTGTAGAAACTAATAATGAACTGTATGAA

CAACTATTGGAATTGATAAGAATAAATCATGGACTGCTTGTCTCAATCGG

TGTTTCTCATCCTGGATTAGAACTTATTAAAAATCTGAGCGATGATTTGA

GAATTGGCTCCACAAAACTTACCGGTGCTGGTGGCGGCGGTTGCTCTTTG

ACTTTGTTACGAAGAGACATTACTCAAGAGCAAATTGACAGCTTCAAAAA

GAAATTGCAAGATGATTTTAGTTACGAGACATTTGAAACAGACTTGGGTG

GGACTGGCTGCTGTTTGTTAAGCGCAAAAAATTTGAATAAAGATCTTAAA

ATCAAATCCCTAGTATTCCAATTATTTGAAAATAAAACTACCACAAAGCA

ACAAATTGACGATCTATTATTGCCAGGAAACACGAATTTACCATGGACTT

CATAAGTCGACCTGCAGGCATGCAAGCTTGGCTGTTTTGGCGGATGAGAG

AAGATTTTCAGCCTGATACAGATTAAATCAGAACGCAGAAGCGGTCTGAT

AAAACAGAATTTGCCTGGCGGCAGTAGCGCGGTGGTCCCACCTGACCCCA

TGCCGAACTCAGAAGTGAAACGCCGTAGCGCCGATGGTAGTGTGGGGTCT

CCCCATGCGAGAGTAGGGAACTGCCAGGCATCAAATAAAACGAAAGGCTC

AGTCGAAAGACTGGGCCTTTCGTTTTATCTGTTGTTTGTCGGTGAACGCT

CTCCTGAGTAGGACAAATCCGCCGGGAGCGGATTTGAACGTTGCGAAGCA

ACGGCCCGGAGGGTGGCGGGCAGGACGCCCGCCATAAACTGCCAGGCATC

AAATTAAGCAGAAGGCCATCCTGACGGATGGCCTTTTTGCGTTTCTTTAA

TTAAATTCAAATATGTATCCGCTCATGAGACAATAACCCTGATAAATGCT

TCAATAATATTGAAAAAGGAAGAGTATGAGTATTCAACATTTCCGTGTCG

CCCTTATTCCCTTTTTTGCGGCATTTTGCCTTCCTGTTTTTGCTCACCCA

GAAACGCTGGTGAAAGTAAAAGATGCTGAAGATCAGTTGGGTGCACGAGT

GGGTTACATCGAACTGGATCTCAACAGCGGTAAGATCCTTGAGAGTTTTC

GCCCCGAAGAACGTTTTCCAATGATGAGCACTTTTAAAGTTCTGCTATGT

GGCGCGGTATTATCCCGTGTTGACGCCGGGCAAGAGCAACTCGGTCGCCG

CATACACTATTCTCAGAATGACTTGGTTGAGTACTCACCAGTCACAGAAA

AGCATCTTACGGATGGCATGACAGTAAGAGAATTATGCAGTGCTGCCATA

ACCATGAGTGATAACACTGCGGCCAACTTACTTCTGACAACGATCGGAGG

ACCGAAGGAGCTAACCGCTTTTTTGCACAACATGGGGGATCATGTAACTC

GCCTTGATCGTTGGGAACCGGAGCTGAATGAAGCCATACCAAACGACGAG

CGTGACACCACGATGCCTACAGCAATGGCAACAACGTTGCGCAAACTATT

AACTGGCGAACTACTTACTCTAGCTTCCCGGCAACAATTAATAGACTGGA

TGGAGGCGGATAAAGTTGCAGGACCACTTCTGCGCTCGGCCCTTCCGGCT

GGCTGGTTTATTGCTGATAAATCTGGAGCCGGTGAGCGTGGGTCTCGCGG

TATCATTGCAGCACTGGGGCCAGATGGTAAGCCCTCCCGTATCGTAGTTA

TCTACACGACGGGGAGTCAGGCAACTATGGATGAACGAAATAGACAGATC

GCTGAGATAGGTGCCTCACTGATTAAGCATTGGTAACTGTCAGACCAAGT

TTACTCATATATACTTTAGATTGATTTAAAACTTCATTTTTAATTTAAAA

GGATCTAGGTGAAGATCCTTTTTGATAATCTCATGACCAAAATCCCTTAA

CGTGAGTTTTCGTTCCACTGAGCGTCAGACCCCGTAGAAAAGATCAAAGG

ATCTTCTTGAGATCCTTTTTTTCTGCGCGTAATCTGCTGCTTGCAAACAA

AAAAACCACCGCTACCAGCGGTGGTTTGTTTGCCGGATCAAGAGCTACCA

ACTCTTTTTCCGAAGGTAACTGGCTTCAGCAGAGCGCAGATACCAAATAC

TGTCCTTCTAGTGTAGCCGTAGTTAGGCCACCACTTCAAGAACTCTGTAG

CACCGCCTACATACCTCGCTCTGCTAATCCTGTTACCAGTGGCTGCTGCC

AGTGGCGATAAGTCGTGTCTTACCGGGTTGGACTCAAGACGATAGTTACC

GGATAAGGCGCAGCGGTCGGGCTGAACGGGGGGTTCGTGCACACAGCCCA

GCTTGGAGCGAACGACCTACACCGAACTGAGATACCTACAGCGTGAGCTA

TGAGAAAGCGCCACGCTTCCCGAAGGGAGAAAGGCGGACAGGTATCCGGT

AAGCGGCAGGGTCGGAACAGGAGAGCGCACGAGGGAGCTTCCAGGGGGAA

ACGCCTGGTATCTTTATAGTCCTGTCGGGTTTCGCCACCTCTGACTTGAG

CGTCGATTTTTGTGATGCTCGTCAGGGGGGCGGAGCCTATGGAAAAACGC

CAGCAACGCGGCCTTTTTACGGTTCCTGGCCTTTTGCTGGCCTTTTGCTC

ACATGTTCTTTCCTGCGTTAATTAACTAGTCATATGGGCATGCATTTACG

TTGACACCATCGAATGGTGCAAAACCTTTCGCGGTATGGCATGATAGCGC

CCGGAAGAGAGTCAATTCAGGGTGGTGAATGTGAAACCAGTAACGTTATA

CGATGTCGCAGAGTATGCCGGTGTCTCTTATCAGACCGTTTCCCGCGTGG

TGAACCAGGCCAGCCACGTTTCTGCGAAAACGCGGGAAAAAGTGGAAGCG

GCGATGGCGGAGCTGAATTACATTCCCAACCGCGTGGCACAACAACTGGC

GGGCAAACAGTCGTTGCTGATTGGCGTTGCCACCTCCAGTCTGGCCCTGC

ACGCGCCGTCGCAAATTGTCGCGGCGATTAAATCTCGCGCCGATCAACTG

GGTGCCAGCGTGGTGGTGTCGATGGTAGAACGAAGCGGCGTCGAAGCCTG

TAAAGCGGCGGTGCACAATCTTCTCGCGCAACGCGTCAGTGGGCTGATCA

TTAACTATCCGCTGGATGACCAGGATGCCATTGCTGTGGAAGCTGCCTGC

ACTAATGTTCCGGCGTTATTTCTTGATGTCTCTGACCAGACACCCATCAA

CAGTATTATTTTCTCCCATGAAGACGGTACGCGACTGGGCGTGGAGCATC

TGGTCGCATTGGGTCACCAGCAAATCGCGCTGTTAGCGGGCCCATTAAGT

TCTGTCTCGGCGCGTCTGCGTCTGGCTGGCTGGCATAAATATCTCACTCG

CAATCAAATTCAGCCGATAGCGGAACGGGAAGGCGACTGGAGTGCCATGT

CCGGTTTTCAACAAACCATGCAAATGCTGAATGAGGGCATCGTTCCCACT

GCGATGCTGGTTGCCAACGATCAGATGGCGCTGGGCGCAATGCGCGCCAT

TACCGAGTCCGGGCTGCGCGTTGGTGCGGATATCTCGGTAGTGGGATACG

ACGATACCGAAGACAGCTCATGTTATATCCCGCCGTTAACCACCATCAAA

CAGGATTTTCGCCTGCTGGGGCAAACCAGCGTGGACCGCTTGCTGCAACT

CTCTCAGGGCCAGGCGGTGAAGGGCAATCAGCTGTTGCCCGTCTCACTGG

TGAAAAGAAAAACCACCCTGGCGCCCAATACGCAAACCGCCTCTCCCCGC

GCGTTGGCCGATTCATTAATGCAGCTGGCACGACAGGTTTCCCGACTGGA

AAGCGGGCAGTGAGCGCAACGCAATTAATGTGAGTTAGCGCGAATTGATC

TG

pACYC184-M-Mmm


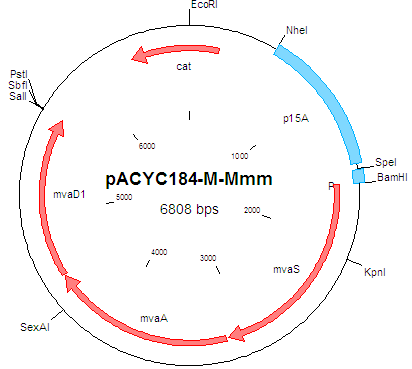


>pACYC184-M-Mmm

GAATTCCGGATGAGCATTCATCAGGCGGGCAAGAATGTGAATAAAGGCCG

GATAAAACTTGTGCTTATTTTTCTTTACGGTCTTTAAAAAGGCCGTAATA

TCCAGCTGAACGGTCTGGTTATAGGTACATTGAGCAACTGACTGAAATGC

CTCAAAATGTTCTTTACGATGCCATTGGGATATATCAACGGTGGTATATC

CAGTGATTTTTTTCTCCATTTTAGCTTCCTTAGCTCCTGAAAATCTCGAT

AACTCAAAAAATACGCCCGGTAGTGATCTTATTTCATTATGGTGAAAGTT

GGAACCTCTTACGTGCCGATCAACGTCTCATTTTCGCCAAAAGTTGGCCC

AGGGCTTCCCGGTATCAACAGGGACACCAGGATTTATTTATTCTGCGAAG

TGATCTTCCGTCACAGGTATTTATTCGGCGCAAAGTGCGTCGGGTGATGC

TGCCAACTTACTGATTTAGTGTATGATGGTGTTTTTGAGGTGCTCCAGTG

GCTTCTGTTTCTATCAGCTGTCCCTCCTGTTCAGCTACTGACGGGGTGGT

GCGTAACGGCAAAAGCACCGCCGGACATCAGCGCTAGCGGAGTGTATACT

GGCTTACTATGTTGGCACTGATGAGGGTGTCAGTGAAGTGCTTCATGTGG

CAGGAGAAAAAAGGCTGCACCGGTGCGTCAGCAGAATATGTGATACAGGA

TATATTCCGCTTCCTCGCTCACTGACTCGCTACGCTCGGTCGTTCGACTG

CGGCGAGCGGAAATGGCTTACGAACGGGGCGGAGATTTCCTGGAAGATGC

CAGGAAGATACTTAACAGGGAAGTGAGAGGGCCGCGGCAAAGCCGTTTTT

CCATAGGCTCCGCCCCCCTGACAAGCATCACGAAATCTGACGCTCAAATC

AGTGGTGGCGAAACCCGACAGGACTATAAAGATACCAGGCGTTTCCCCCT

GGCGGCTCCCTCGTGCGCTCTCCTGTTCCTGCCTTTCGGTTTACCGGTGT

CATTCCGCTGTTATGGCCGCGTTTGTCTCATTCCACGCCTGACACTCAGT

TCCGGGTAGGCAGTTCGCTCCAAGCTGGACTGTATGCACGAACCCCCCGT

TCAGTCCGACCGCTGCGCCTTATCCGGTAACTATCGTCTTGAGTCCAACC

CGGAAAGACATGCAAAAGCACCACTGGCAGCAGCCACTGGTAATTGATTT

AGAGGAGTTAGTCTTGAAGTCATGCGCCGGTTAAGGCTAAACTGAAAGGA

CAAGTTTTGGTGACTGCGCTCCTCCAAGCCAGTTACCTCGGTTCAAAGAG

TTGGTAGCTCAGAGAACCTTCGAAAAACCGCCCTGCAAGGCGGTTTTTTC

GTTTTCAGAGCAAGAGATTACGCGCAGACCAAAACGATCTCAAGAAGATC

ATCTTATTAATCAGATAAAATATTTCTAGATTTCAGTGCAATTTATCTCT

TCAAATGTAGCACCTGAAGTCAGCCCCATACGATATAAGTTGTAATTCTC

ATGTTTGACAGCTTATCATCGTTAATTAACTAGTCATATGTTATCTCTGG

CGGTGTTGACAAGAGATAACAACGTTGATATAATTGAGCCAATTGTGAGC

GGATAACAATTTCACACGGATCCAGGAGGATTACTATATGAAACTCTCAA

CTAAACTTTGTTGGTGTGGTATTAAAGGAAGACTTAGGCCGCAAAAGCAA

CAACAATTACACAATACAAACTTGCAAATGACTGAACTAAAAAAACAAAA

GACCGCTGAACAAAAAACCAGACCTCAAAATGTCGGTATTAAAGGTATCC

AAATTTACATCCCAACTCAATGTGTCAACCAATCTGAGCTAGAGAAATTT

GATGGCGTTTCTCAAGGTAAATACACAATTGGTCTGGGCCAAACCAACAT

GTCTTTTGTCAATGACAGAGAAGATATCTACTCGATGTCCCTAACTGTTT

TGTCTAAGTTGATCAAGAGTTACAACATCGACACCAACAAAATTGGTAGA

TTAGAAGTCGGTACTGAAACTCTGATTGACAAGTCCAAGTCTGTCAAGTC

TGTCTTGATGCAATTGTTTGGTGAAAACACTGACGTCGAAGGTATTGACA

CGCTTAATGCCTGTTACGGTGGTACCAACGCGTTGTTCAACTCTTTGAAC

TGGATTGAATCTAACGCATGGGATGGTAGAGACGCCATTGTAGTTTGCGG

TGATATTGCCATCTACGATAAGGGTGCCGCAAGACCAACCGGTGGTGCCG

GTACTGTTGCTATGTGGATCGGTCCTGATGCTCCAATTGTATTTGACTCT

GTAAGAGCTTCTTACATGGAACACGCCTACGATTTTTACAAGCCAGATTT

CACCAGCGAATATCCTTACGTCGATGGTCATTTTTCATTAACTTGTTACG

TCAAGGCTCTTGATCAAGTTTACAAGAGTTATTCCAAGAAGGCTATTTCT

AAAGGGTTGGTTAGCGATCCCGCTGGTTCGGATGCTTTGAACGTTTTGAA

ATATTTCGACTACAACGTTTTCCATGTTCCAACCTGTAAATTGGTCACAA

AATCATACGGTAGATTACTATATAACGATTTCAGAGCCAATCCTCAATTG

TTCCCAGAAGTTGACGCCGAATTAGCTACTCGCGATTATGACGAATCTTT

AACCGATAAGAACATTGAAAAAACTTTTGTTAATGTTGCTAAGCCATTCC

ACAAAGAGAGAGTTGCCCAATCTTTGATTGTTCCAACAAACACAGGTAAC

ATGTACACCGCATCTGTTTATGCCGCCTTTGCATCTCTATTAAACTATGT

TGGATCTGACGACTTACAAGGCAAGCGTGTTGGTTTATTTTCTTACGGTT

CCGGTTTAGCTGCATCTCTATATTCTTGCAAAATTGTTGGTGACGTCCAA

CATATTATCAAGGAATTAGATATTACTAACAAATTAGCCAAGAGAATCAC

CGAAACTCCAAAGGATTACGAAGCTGCCATCGAATTGAGAGAAAATGCCC

ATTTGAAGAAGAACTTCAAACCTCAAGGTTCCATTGAGCATTTGCAAAGT

GGTGTTTACTACTTGACCAACATCGATGACAAATTTAGAAGATCTTACGA

TGTTAAAAAATAGGAGGCAGATCAAATGTCAGAGTTGAGAGCCTTCAGTG

CCCCAGGGAAAGCGTTACTAGCTGGTGGATATTTAGTTTTAGATACAAAA

TATGAAGCATTTGTAGTCGGATTATCGGCAAGAATGCATGCTGTAGCCCA

TCCTTACGGTTCATTGCAAGGGTCTGATAAGTTTGAAGTGCGTGTGAAAA

GTAAACAATTTAAAGATGGGGAGTGGCTGTACCATATAAGTCCTAAAAGT

GGCTTCATTCCTGTTTCGATAGGCGGATCTAAGAACCCTTTCATTGAAAA

AGTTATCGCTAACGTATTTAGCTACTTTAAACCTAACATGGACGACTACT

GCAATAGAAACTTGTTCGTTATTGATATTTTCTCTGATGATGCCTACCAT

TCTCAGGAGGATAGCGTTACCGAACATCGTGGCAACAGAAGATTGAGTTT

TCATTCGCACAGAATTGAAGAAGTTCCCAAAACAGGGCTGGGCTCCTCGG

CAGGTTTAGTCACAGTTTTAACTACAGCTTTGGCCTCCTTTTTTGTATCG

GACCTGGAAAATAATGTAGACAAATATAGAGAAGTTATTCATAATTTAGC

ACAAGTTGCTCATTGTCAAGCTCAGGGTAAAATTGGAAGCGGGTTTGATG

TAGCGGCGGCAGCATATGGATCTATCAGATATAGAAGATTCCCACCCGCA

TTAATCTCTAATTTGCCAGATATTGGAAGTGCTACTTACGGCAGTAAACT

GGCGCATTTGGTTGATGAAGAAGACTGGAATATTACGATTAAAAGTAACC

ATTTACCTTCGGGATTAACTTTATGGATGGGCGATATTAAGAATGGTTCA

GAAACAGTAAAACTGGTCCAGAAGGTAAAAAATTGGTATGATTCGCATAT

GCCAGAAAGCTTGAAAATATATACAGAACTCGATCATGCAAATTCTAGAT

TTATGGATGGACTATCTAAACTAGATCGCTTACACGAGACTCATGACGAT

TACAGCGATCAGATATTTGAGTCTCTTGAGAGGAATGACTGTACCTGTCA

AAAGTATCCTGAAATCACAGAAGTTAGAGATGCAGTTGCCACAATTAGAC

GTTCCTTTAGAAAAATAACTAAAGAATCTGGTGCCGATATCGAACCTCCC

GTACAAACTAGCTTATTGGATGATTGCCAGACCTTAAAAGGAGTTCTTAC

TTGCTTAATACCTGGTGCTGGTGGTTATGACGCCATTGCAGTGATTACTA

AGCAAGATGTTGATCTTAGGGCTCAAACCGCTAATGACAAAAGATTTTCT

AAGGTTCAATGGCTGGATGTAACTCAGGCTGACTGGGGTGTTAGGAAAGA

AAAAGATCCGGAAACTTATCTTGATAAATAGGAGGTAATACTCATGACCG

TTTACACAGCATCCGTTACCGCACCCGTCAACATCGCAACCCTTAAGTAT

TGGGGGAAAAGGGACACGAAGTTGAATCTGCCCACCAATTCGTCCATATC

AGTGACTTTATCGCAAGATGACCTCAGAACGTTGACCTCTGCGGCTACTG

CACCTGAGTTTGAACGCGACACTTTGTGGTTAAATGGAGAACCACACAGC

ATCGACAATGAAAGAACTCAAAATTGTCTGCGCGACCTACGCCAATTAAG

AAAGGAAATGGAATCGAAGGACGCCTCATTGCCCACATTATCTCAATGGA

AACTCCACATTGTCTCCGAAAATAACTTTCCTACAGCAGCTGGTTTAGCT

TCCTCCGCTGCTGGCTTTGCTGCATTGGTCTCTGCAATTGCTAAGTTATA

CCAATTACCACAGTCAACTTCAGAAATATCTAGAATAGCAAGAAAGGGGT

CTGGTTCAGCTTGTAGATCGTTGTTTGGCGGATACGTGGCCTGGGAAATG

GGAAAAGCTGAAGATGGTCATGATTCCATGGCAGTACAAATCGCAGACAG

CTCTGACTGGCCTCAGATGAAAGCTTGTGTCCTAGTTGTCAGCGATATTA

AAAAGGATGTGAGTTCCACTCAGGGTATGCAATTGACCGTGGCAACCTCC

GAACTATTTAAAGAAAGAATTGAACATGTCGTACCAAAGAGATTTGAAGT

CATGCGTAAAGCCATTGTTGAAAAAGATTTCGCCACCTTTGCAAAGGAAA

CAATGATGGATTCCAACTCTTTCCATGCCACATGTTTGGACTCTTTCCCT

CCAATATTCTACATGAATGACACTTCCAAGCGTATCATCAGTTGGTGCCA

CACCATTAATCAGTTTTACGGAGAAACAATCGTTGCATACACGTTTGATG

CAGGTCCAAATGCTGTGTTGTACTACTTAGCTGAAAATGAGTCGAAACTC

TTTGCATTTATCTATAAATTGTTTGGCTCTGTTCCTGGATGGGACAAGAA

ATTTACTACTGAGCAGCTTGAGGCTTTCAACCATCAATTTGAATCATCTA

ACTTTACTGCACGTGAATTGGATCTTGAGTTGCAAAAGGATGTTGCCAGA

GTGATTTTAACTCAAGTCGGTTCAGGCCCACAAGAAACAAACGAATCTTT

GATTGACGCAAAGACTGGTCTACCAAAGGAATAAGTCGACCTGCAGGCAT

GCAAGCTTGGCTGTTTTGGCGGATGAGAGAAGATTTTCAGCCTGATACAG

ATTAAATCAGAACGCAGAAGCGGTCTGATAAAACAGAATTTGCCTGGCGG

CAGTAGCGCGGTGGTCCCACCTGACCCCATGCCGAACTCAGAAGTGAAAC

GCCGTAGCGCCGATGGTAGTGTGGGGTCTCCCCATGCGAGAGTAGGGAAC

TGCCAGGCATCAAATAAAACGAAAGGCTCAGTCGAAAGACTGGGCCTTTC

GTTTTATCTGTTGTTTGTCGGTGAACGCTCTCCTGAGTAGGACAAATCCG

CCGGGAGCGGATTTGAACGTTGCGAAGCAACGGCCCGGAGGGTGGCGGGC

AGGACGCCCGCCATAAACTGCCAGGCATCAAATTAAGCAGAAGGCCATCC

TGACGGATGGCCTTTTTGCGTTTCTTTAATTAAGGGAGAGCCTGAGCAAA

CTGGCCTCAGGCATTTGAGAAGCACACGGTCACACTGCTTCCGGTAGTCA

ATAAACCGGTAAACCAGCAATAGACATAAGCGGCTATTTAACGACCCTGC

CCTGAACCGACGACCGGGTCGAATTTGCTTTCGAATTTCTGCCATTCATC

CGCTTATTATCACTTATTCAGGCGTAGCACCAGGCGTTTAAGGGCACCAA

TAACTGCCTTAAAAAAATTACGCCCCGCCCTGCCACTCATCGCAGTACTG

TTGTAATTCATTAAGCATTCTGCCGACATGGAAGCCATCACAGACGGCAT

GATGAACCTGAATCGCCAGCGGCATCAGCACCTTGTCGCCTTGCGTATAA

TATTTGCCCATGGTGAAAACGGGGGCGAAGAAGTTGTCCATATTGGCCAC

GTTTAAATCAAAACTGGTGAAACTCACCCAGGGATTGGCTGAGACGAAAA

ACATATTCTCAATAAACCCTTTAGGGAAATAGGCCAGGTTTTCACCGTAA

CACGCCACATCTTGCGAATATATGTGTAGAAACTGCCGGAAATCGTCGTG

GTATTCACTCCAGAGCGATGAAAACGTTTCAGTTTGCTCATGGAAAACGG

TGTAACAAGGGTGAACACTATCCCATATCACCAGCTCACCGTCTTTCATT

GCCATACG

pQL003-He

>pQL003-He Ligation of He into pflB

AGCGCCCAATACGCAAACCGCCTCTCCCCGCGCGTTGGCCGATTCATTAA

TGCAGCTGGCACGACAGGTTTCCCGACTGGAAAGCGGGCAGTGAGCGCAA

CGCAATTAATGTGAGTTAGCTCACTCATTAGGCACCCCAGGCTTTACACT

TTATGCTTCCGGCTCGTATGTTGTGTGGAATTGTGAGCGGATAACAATTT

CACACAGGAAACAGCTATGACCATGATTACGCCAAGCTTGGTACCGAGCT

CGGATCCACTAGTAACGGCCGCCAGTGTGCTGGAATTCGCCCTTTGTCCG

AGCTTAATGAAAAGTTAGCCACAGCCTGGGAAGGTTTTACCAAAGGTGAC

TGGCAGAATGAAGTAAACGTCCGTGACTTCATTCAGAAAAACTACACTCC

GTACGAGGGTGACGAGTCCTTCCTGGCTGGCGCTACTGAAGCGACCACCA

CCCTGTGGGACAAAGTAATGGAAGGCGTTAAACTGGAAAACCGCACTCAC

GCGCCAGTTGACTTTGACACCGCTGTTGCTTCCACCATCACCTCTCACGA

CGCTGGCTACATCAACAAGCAGCTTGAGAAAATCGTTGGTCTGCAGACTG

AAGCTCCGCTGAAACGTGCTCTTATCCCGTTCGGTGGTATCAAAATGATC

GAAGGTTCCTGCAAAGCGTACAACCGCGAACTGGATCCGATGATCAAAAA

AATCTTCACTGAATACCGTAAAACTCACAACCAGGGCGTGTTCGACGTTT

ACACTCCGCCCGGGAAGGAGATATACCATGGTTTTAACCAATAAAACAGT

CATTTCTGGATCGAAAGTCAAAAGTTTATCATCTGCGCAATCGAGCTCAT

CAGGACCTTCATCATCTAGTGAGGAAGATGATTCCCGCGATATTGAAAGC

TTGGATAAGAAAATACGTCCTTTAGAAGAATTAGAAGCATTATTAAGTAG

TGGAAATACAAAACAATTGAAGAACAAAGAGGTCGCTGCCTTGGTTATTC

ACGGTAAGTTACCTTTGTACGCTTTGGAGAAAAAATTAGGTGATACTACG

AGAGCGGTTGCGGTACGTAGGAAGGCTCTTTCAATTTTGGCAGAAGCTCC

TGTATTAGCATCTGATCGTTTACCATATAAAAATTATGACTACGACCGCG

TATTTGGCGCTTGTTGTGAAAATGTTATAGGTTACATGCCTTTGCCCGTT

GGTGTTATAGGCCCCTTGGTTATCGATGGTACATCTTATCATATACCAAT

GGCAACTACAGAGGGTTGTTTGGTAGCTTCTGCCATGCGTGGCTGTAAGG

CAATCAATGCTGGCGGTGGTGCAACAACTGTTTTAACTAAGGATGGTATG

ACAAGAGGCCCAGTAGTCCGTTTCCCAACTTTGAAAAGATCTGGTGCCTG

TAAGATATGGTTAGACTCAGAAGAGGGACAAAACGCAATTAAAAAAGCTT

TTAACTCTACATCAAGATTTGCACGTCTGCAACATATTCAAACTTGTCTA

GCAGGAGATTTACTCTTCATGAGATTTAGAACAACTACTGGTGACGCAAT

GGGTATGAATATGATTTCTAAAGGTGTCGAATACTCATTAAAGCAAATGG

TAGAAGAGTATGGCTGGGAAGATATGGAGGTTGTCTCCGTTTCTGGTAAC

TACTGTACCGACAAAAAACCAGCTGCCATCAACTGGATCGAAGGTCGTGG

TAAGAGTGTCGTCGCAGAAGCTACTATTCCTGGTGATGTTGTCAGAAAAG

TGTTAAAAAGTGATGTTTCCGCATTGGTTGAGTTGAACATTGCTAAGAAT

TTGGTTGGATCTGCAATGGCTGGGTCTGTTGGTGGATTTAACGCACATGC

AGCTAATTTAGTGACAGCTGTTTTCTTGGCATTAGGACAAGATCCTGCAC

AAAATGTTGAAAGTTCCAACTGTATAACATTGATGAAAGAAGTGGACGGT

GATTTGAGAATTTCCGTATCCATGCCATCCATCGAAGTAGGTACCATCGG

TGGTGGTACTGTTCTAGAACCACAAGGTGCCATGTTGGACTTATTAGGTG

TAAGAGGCCCGCATGCTACCGCTCCTGGTACCAACGCACGTCAATTAGCA

AGAATAGTTGCCTGTGCCGTCTTGGCAGGTGAATTATCCTTATGTGCTGC

CCTAGCAGCCGGCCATTTGGTTCAAAGTCATATGACCCACAACAGGAAAC

CTGCTGAACCAACAAAACCTAACAATTTGGACGCCACTGATATAAATCGT

TTGAAAGATGGGTCCGTCACCTGCATTAAATCCTAGGAGATATACCATGT

CATTACCGTTCTTAACTTCTGCACCGGGAAAGGTTATTATTTTTGGTGAA

CACTCTGCTGTGTACAACAAGCCTGCCGTCGCTGCTAGTGTGTCTGCGTT

GAGAACCTACCTGCTAATAAGCGAGTCATCTGCACCAGATACTATTGAAT

TGGACTTCCCGGACATTAGCTTTAATCATAAGTGGTCCATCAATGATTTC

AATGCCATCACCGAGGATCAAGTAAACTCCCAAAAATTGGCCAAGGCTCA

ACAAGCCACCGATGGCTTGTCTCAGGAACTCGTTAGTCTTTTGGATCCGT

TGTTAGCTCAACTATCCGAATCCTTCCACTACCATGCAGCGTTTTGTTTC

CTGTATATGTTTGTTTGCCTATGCCCCCATGCCAAGAATATTAAGTTTTC

TTTAAAGTCTACTTTACCCATCGGTGCTGGGTTGGGCTCAAGCGCCTCTA

TTTCTGTATCACTGGCCTTAGCTATGGCCTACTTGGGGGGGTTAATAGGA

TCTAATGACTTGGAAAAGCTGTCAGAAAACGATAAGCATATAGTGAATCA

ATGGGCCTTCATAGGTGAAAAGTGTATTCACGGTACCCCTTCAGGAATAG

ATAACGCTGTGGCCACTTATGGTAATGCCCTGCTATTTGAAAAAGACTCA

CATAATGGAACAATAAACACAAACAATTTTAAGTTCTTAGATGATTTCCC

AGCCATTCCAATGATCCTAACCTATACTAGAATTCCAAGGTCTACAAAAG

ATCTTGTTGCTCGCGTTCGTGTGTTGGTCACCGAGAAATTTCCTGAAGTT

ATGAAGCCAATTCTAGATGCCATGGGTGAATGTGCCCTACAAGGCTTAGA

GATCATGACTAAGTTAAGTAAATGTAAAGGCACCGATGACGAGGCTGTAG

AAACTAATAATGAACTGTATGAACAACTATTGGAATTGATAAGAATAAAT

CATGGACTGCTTGTCTCAATCGGTGTTTCTCATCCTGGATTAGAACTTAT

TAAAAATCTGAGCGATGATTTGAGAATTGGCTCCACAAAACTTACCGGTG

CTGGTGGCGGCGGTTGCTCTTTGACTTTGTTACGAAGAGACATTACTCAA

GAGCAAATTGACAGCTTCAAAAAGAAATTGCAAGATGATTTTAGTTACGA

GACATTTGAAACAGACTTGGGTGGGACTGGCTGCTGTTTGTTAAGCGCAA

AAAATTTGAATAAAGATCTTAAAATCAAATCCCTAGTATTCCAATTATTT

GAAAATAAAACTACCACAAAGCAACAAATTGACGATCTATTATTGCCAGG

AAACACGAATTTACCATGGACTTCATAAGTCGACCTGCAGGCATGCAAGC

TTGGCTGTTTTGGCGGATGAGAGAAGATTTTCAGCCTGATACAGATTAAA

TCAGAACGCAGAAGCGGTCTGATAAAACAGAATTTGCCTGGCGGCAGTAG

CGCGGTGGTCCCACCTGACCCCATGCCGAACTCAGAAGTGAAACGCCGTA

GCGCCGATGGTAGTGTGGGGTCTCCCCATGCGAGAGTAGGGAACTGCCAG

GCATCAAATAAAACGAAAGGCTCAGTCGAAAGACTGGGCCTTTCGTTTTA

TCTGTTGTTTGTCGGTGAACGCTCTCCTGAGTAGGACAAATCCGCCGGGA

GCGGATTTGAACGTTGCGAAGCAACGGCCCGGAGGGTGGCGGGCAGGACG

CCCGCCATAAACTGCCAGGCATCAAATTAAGCAGAAGGCCATCCTGACGG

ATGGCCTTTTTGCGTTTCTAAACGGGTAACACCCCAGACGGTCGTCGTGC

TGGCGCGCCGTTCGGACCGGGTGCTAACCCGATGCACGGTCGTGACCAGA

AAGGTGCAGTAGCCTCTCTGACTTCCGTTGCTAAACTGCCGTTTGCTTAC

GCTAAAGATGGTATCTCCTACACCTTCTCTATCGTTCCGAACGCACTGGG

TAAAGACGACGAAGTTCGTAAGACCAACCTGGCTGGTCTGATGGATGGTT

ACTTCCACCACGAAGCATCCATCGAAGGTGGTCAGCACCTGAACGTTAAC

GTGATGAACCGTGAAATGCTGCTCGACGCGATGGAAAACCCGGAAAAATA

TCCGCAGCTGACCATCCGTGTATCTGGCTACGCAGTACGTTTCAACTCGC

TGACTAAAGAACAGCAGCAGGACGTTATTACTCGAAGGGCGAATTCTGCA

GATATCCATCACACTGGCGGCCGCTCGAGCATGCATCTAGAGGGCCCAAT

TCGCCCTATAGTGAGTCGTATTACAATTCACTGGCCGTCGTTTTACAACG

TCGTGACTGGGAAAACCCTGGCGTTACCCAACTTAATCGCCTTGCAGCAC

ATCCCCCTTTCGCCAGCTGGCGTAATAGCGAAGAGGCCCGCACCGATCGC

CCTTCCCAACAGTTGCGCAGCCTGAATGGCGAATGGACGCGCCCTGTAGC

GGCGCATTAAGCGCGGCGGGTGTGGTGGTTACGCGCAGCGTGACCGCTAC

ACTTGCCAGCGCCCTAGCGCCCGCTCCTTTCGCTTTCTTCCCTTCCTTTC

TCGCCACGTTCGCCGGCTTTCCCCGTCAAGCTCTAAATCGGGGGCTCCCT

TTAGGGTTCCGATTTAGTGCTTTACGGCACCTCGACCCCAAAAAACTTGA

TTAGGGTGATGGTTCACGTAGTGGGCCATCGCCCTGATAGACGGTTTTTC

GCCCTTTGACGTTGGAGTCCACGTTCTTTAATAGTGGACTCTTGTTCCAA

ACTGGAACAACACTCAACCCTATCTCGGTCTATTCTTTTGATTTATAAGG

GATTTTGCCGATTTCGGCCTATTGGTTAAAAAATGAGCTGATTTAACAAA

AATTTAACGCGAATTTTAACAAAATTCAGGGCGCAAGGGCTGCTAAAGGA

AGCGGAACACGTAGAAAGCCAGTCCGCAGAAACGGTGCTGACCCCGGATG

AATGTCAGCTACTGGGCTATCTGGACAAGGGAAAACGCAAGCGCAAAGAG

AAAGCAGGTAGCTTGCAGTGGGCTTACATGGCGATAGCTAGACTGGGCGG

TTTTATGGACAGCAAGCGAACCGGAATTGCCAGCTGGGGCGCCCTCTGGT

AAGGTTGGGAAGCCCTGCAAAGTAAACTGGATGGCTTTCTTGCCGCCAAG

GATCTGATGGCGCAGGGGATCAAGATCTGATCAAGAGACAGGATGAGGAT

CGTTTCGCATGATTGAACAAGATGGATTGCACGCAGGTTCTCCGGCCGCT

TGGGTGGAGAGGCTATTCGGCTATGACTGGGCACAACAGACAATCGGCTG

CTCTGATGCCGCCGTGTTCCGGCTGTCAGCGCAGGGGCGCCCGGTTCTTT

TTGTCAAGACCGACCTGTCCGGTGCCCTGAATGAACTGCAGGACGAGGCA

GCGCGGCTATCGTGGCTGGCCACGACGGGCGTTCCTTGCGCAGCTGTGCT

CGACGTTGTCACTGAAGCGGGAAGGGACTGGCTGCTATTGGGCGAAGTGC

CGGGGCAGGATCTCCTGTCATCCCACCTTGCTCCTGCCGAGAAAGTATCC

ATCATGGCTGATGCAATGCGGCGGCTGCATACGCTTGATCCGGCTACCTG

CCCATTCGACCACCAAGCGAAACATCGCATCGAGCGAGCACGTACTCGGA

TGGAAGCCGGTCTTGTCGATCAGGATGATCTGGACGAAGAGCATCAGGGG

CTCGCGCCAGCCGAACTGTTCGCCAGGCTCAAGGCGCGCATGCCCGACGG

CGAGGATCTCGTCGTGACCCATGGCGATGCCTGCTTGCCGAATATCATGG

TGGAAAATGGCCGCTTTTCTGGATTCATCGACTGTGGCCGGCTGGGTGTG

GCGGACCGCTATCAGGACATAGCGTTGGCTACCCGTGATATTGCTGAAGA

GCTTGGCGGCGAATGGGCTGACCGCTTCCTCGTGCTTTACGGTATCGCCG

CTCCCGATTCGCAGCGCATCGCCTTCTATCGCCTTCTTGACGAGTTCTTC

TGAATTGAAAAAGGAAGAGTATGAGTATTCAACATTTCCGTGTCGCCCTT

ATTCCCTTTTTTGCGGCATTTTGCCTTCCTGTTTTTGCTCACCCAGAAAC

GCTGGTGAAAGTAAAAGATGCTGAAGATCAGTTGGGTGCACGAGTGGGTT

ACATCGAACTGGATCTCAACAGCGGTAAGATCCTTGAGAGTTTTCGCCCC

GAAGAACGTTTTCCAATGATGAGCACTTTTAAAGTTCTGCTATGTGGCGC

GGTATTATCCCGTATTGACGCCGGGCAAGAGCAACTCGGTCGCCGCATAC

ACTATTCTCAGAATGACTTGGTTGAGTACTCACCAGTCACAGAAAAGCAT

CTTACGGATGGCATGACAGTAAGAGAATTATGCAGTGCTGCCATAACCAT

GAGTGATAACACTGCGGCCAACTTACTTCTGACAACGATCGGAGGACCGA

AGGAGCTAACCGCTTTTTTGCACAACATGGGGGATCATGTAACTCGCCTT

GATCGTTGGGAACCGGAGCTGAATGAAGCCATACCAAACGACGAGCGTGA

CACCACGATGCCTGTAGCAATGGCAACAACGTTGCGCAAACTATTAACTG

GCGAACTACTTACTCTAGCTTCCCGGCAACAATTAATAGACTGGATGGAG

GCGGATAAAGTTGCAGGACCACTTCTGCGCTCGGCCCTTCCGGCTGGCTG

GTTTATTGCTGATAAATCTGGAGCCGGTGAGCGTGGGTCTCGCGGTATCA

TTGCAGCACTGGGGCCAGATGGTAAGCCCTCCCGTATCGTAGTTATCTAC

ACGACGGGGAGTCAGGCAACTATGGATGAACGAAATAGACAGATCGCTGA

GATAGGTGCCTCACTGATTAAGCATTGGTAACTGTCAGACCAAGTTTACT

CATATATACTTTAGATTGATTTAAAACTTCATTTTTAATTTAAAAGGATC

TAGGTGAAGATCCTTTTTGATAATCTCATGACCAAAATCCCTTAACGTGA

GTTTTCGTTCCACTGAGCGTCAGACCCCGTAGAAAAGATCAAAGGATCTT

CTTGAGATCCTTTTTTTCTGCGCGTAATCTGCTGCTTGCAAACAAAAAAA

CCACCGCTACCAGCGGTGGTTTGTTTGCCGGATCAAGAGCTACCAACTCT

TTTTCCGAAGGTAACTGGCTTCAGCAGAGCGCAGATACCAAATACTGTTC

TTCTAGTGTAGCCGTAGTTAGGCCACCACTTCAAGAACTCTGTAGCACCG

CCTACATACCTCGCTCTGCTAATCCTGTTACCAGTGGCTGCTGCCAGTGG

CGATAAGTCGTGTCTTACCGGGTTGGACTCAAGACGATAGTTACCGGATA

AGGCGCAGCGGTCGGGCTGAACGGGGGGTTCGTGCACACAGCCCAGCTTG

GAGCGAACGACCTACACCGAACTGAGATACCTACAGCGTGAGCTATGAGA

AAGCGCCACGCTTCCCGAAGGGAGAAAGGCGGACAGGTATCCGGTAAGCG

GCAGGGTCGGAACAGGAGAGCGCACGAGGGAGCTTCCAGGGGGAAACGCC

TGGTATCTTTATAGTCCTGTCGGGTTTCGCCACCTCTGACTTGAGCGTCG

ATTTTTGTGATGCTCGTCAGGGGGGCGGAGCCTATGGAAAAACGCCAGCA

ACGCGGCCTTTTTACGGTTCCTGGCCTTTTGCTGGCCTTTTGCTCACATG

TTCTTTCCTGCGTTATCCCCTGATTCTGTGGATAACCGTATTACCGCCTT

TGAGTGAGCTGATACCGCTCGCCGCAGCCGAACGACCGAGCGCAGCGAGT

CAGTGAGCGAGGAAGCGGAAG

pXZ006C

>pXZ006C

AGCGCCCAATACGCAAACCGCCTCTCCCCGCGCGTTGGCCGATTCATTAA

TGCAGCTGGCACGACAGGTTTCCCGACTGGAAAGCGGGCAGTGAGCGCAA

CGCAATTAATGTGAGTTAGCTCACTCATTAGGCACCCCAGGCTTTACACT

TTATGCTTCCGGCTCGTATGTTGTGTGGAATTGTGAGCGGATAACAATTT

CACACAGGAAACAGCTATGACCATGATTACGCCAAGCTTGGTACCGAGCT

CGGATCCACTAGTAACGGCCGCCAGTGTGCTGGAATTCGCCCTTTGCAGA

AAACCATCGACAAGCTGGCAGAGCTGCAGGAACGCTTCAAGCGCGTGCGC

ATCACCGACACTTCCAGCGTGTTCAACACCGACCTGCTCTACACCATTGA

ACTGGGCCACGGTCTGAACGTTGCTGAATGTATGGCGCACTCCGCAATGG

CACGTAAAGAGTCCCGCGGCGCGCACCAGCGTCTGGACGAAGGTTGCACC

GAGCGTGACGACGTCAACTTCCTCAAACACACCCTCGCCTTCCGCGATGC

TGATGGCACGACTCGCCTGGAGTACAGCGACGTGAAGATTACTACGCTGC

CGCCAGCTAAACGCGTTTACGGTGGCGAAGCGGATGCAGCCGATAAGGCG

GAAGCAGCCAATAAGAAGGAGAAGGCGAATGGCTGAGATGAAAAACCTGA

AAATTGAGGTGGTGCGCTATGCCTAATTAATTAATGGATCCATCAAAGGG

AAAACTGTCCATATGCACAGATGAAAACGGTGTAAAAAAGATAGATACAT

CAGAGCTTTTACGAGTTTTTGGTGCATTTAAAGCTGTTCACCATGAACAG

ATCGACAATGTAACAGATGAACAGCATGTAACACCTAATAGAACAGGTGA

AACCAGTAAAACAAAGCAACTAGAACATGAAATTGAACACCTGAGACAAC

TTGTTACAGCTCAACAGTCACACATAGACAGCCTGAGGCATTTGAGAAGC

ACACGGTCACACTGCTTCCGGTAGTCAATAAACCGGNNATCGGCATTTTC

TTTTGCGTTTTTATTTGTTAACTGTTAATTGTCCTTGTTCAAGGATGCTG

TCTTTGACAACAGATGTTTTCTTGCCTTTGATGTTCAGCAGGAAGCTTGG

CGCAAACGTTGATTGTTTGTCTGCGTAGAATCCTCTGTTTGTCATATAGC

TTGTAATCACGACATTGTTTCCTTTCGCTTGAGGTACAGCGAAGTGTGAG

TAAGTAAAGGTTACATCGTTAGGATCAAGATCCATTTTTAACACAAGGCC

AGTTTTGTTCAGCGGCTTGTATGGGCCAGTTAAAGAATTAGAAACATAAC

CAAGCATGTAAATATCGTTAGACGTAATGCCGTCAATCGTCATTTTTGAT

CCGCGGGAGTCAGTGAACAGGTACCATTTGCCGTTCATTTTAAAGACGTT

CGCGCGTTCAATTTCATCTGTTACTGTGTTAGATGCAATCAGCGGTTTCA

TCACTTTTTTCAGTGTGTAATCATCGTTTAGCTCAATCATACCGAGAGCG

CCGTTTGCTAACTCAGCCGTGCGTTTTTTATCGCTTTGCAGAAGTTTTTG

ACTTTCTTGACGGAAGAATGATGTGCTTTTGCCATAGTATGCTTTGTTAA

ATAAAGATTCTTCGCCTTGGTAGCCATCTTCAGTTCCAGTGTTTGCTTCA

AATACTAAGTATTTGTGGCCTTTATCTTCTACGTAGTGAGGATCTCTCAG

CGTATGGTTGTCGCCTGAGCTGTAGTTGCCTTCATCGATGAACTGCTGTA

CATTTTGATACGTTTTTCCGTCACCGTCAAAGATTGATTTATAATCCTCT

ACACCGTTGATGTTCAAAGAGCTGTCTGATGCTGATACGTTAACTTGTGC

AGTTGTCAGTGTTTGTTTGCCGTAATGTTTACCGGAGAAATCAGTGTAGA

ATAAACGGATTTTTCCGTCAGATGTAAATGTGGCTGAACCTGACCATTCT

TGTGTTTGGTCTTTTAGGATAGAATCATTTGCATCGAATTTGTCGCTGTC

TTTAAAGACGCGGCCAGCGTTTTTCCAGCTGTCAATAGAAGTTTCGCCGA

CTTTTTGATAGAACATGTAAATCGATGTGTCATCCGCATTTTTAGGATCT

CCGGCTAATGCAAAGACGATGTGGTAGCCGTGATAGTTTGCGACAGTGCC

GTCAGCGTTTTGTAATGGCCAGCTGTCCCAAACGTCCAGGCCTTTTGCAG

AAGAGATATTTTTAATTGTGGACGAATCGAATTCAGGAACTTGATATTTT

TCATTTTTTTGCTGTTCAGGGATTTGCAGCATATCATGGCGTGTAATATG

GGAAATGCCGTATGTTTCCTTATATGGCTTTTGGTTCGTTTCTTTCGCAA

ACGCTTGAGTTGCGCCTCCTGCCAGCAGTGCGGTAGTAAAGGTTAATACT

GTTGCTTGTTTTGCAAACTTTTTGATGTTCATCGTTCATGTCTCCTTTTT

TATGTACTGTGTTAGCGGTCTGCTTCTTCCAGCCCTCCTGTTTGAAGATG

GCAAGTTAGTTACGCACAATAAAAAAAGACCTAAAATATGTAAGGGGTGA

CGCCAAAGTATACACTTTGCCCTTTACACATTTTAGGTCTTGCCTGCTTT

ATCAGTAACAAACCCGCGCGATTTACTTAGATCTAGCGGCTATTTAACGA

CCCTGCCCTGAACCGACGACCGGGTCGAATTTGCTTTCGAATTTCTGCCA

TTCATCCGCTTATTATCACTTATTCAGGCGTAGCACCAGGCGTTTAAGGG

CACCAATAACTGCCTTAAAAAAATTACGCCCCGCCCTGCCACTCATCGCA

GTACTGTTGTAATTCATTAAGCATTCTGCCGACATGGAAGCCATCACAAA

CGGCATGATGAACCTGAATCGCCAGCGGCATCAGCACCTTGTCGCCTTGC

GTATAATATTTGCCCATGGTGAAAACGGGGGCGAAGAAGTTGTCCATATT

GGCCACGTTTAAATCAAAACTGGTGAAACTCACCCAGGGATTGGCTGAGA

CGAAAAACATATTCTCAATAAACCCTTTAGGGAAATAGGCCAGGTTTTCA

CCGTAACACGCCACATCTTGCGAATATATGTGTAGAAACTGCCGGAAATC

GTCGTGGTATTCACTCCAGAGCGATGAAAACGTTTCAGTTTGCTCATGGA

AAACGGTGTAACAAGGGTGAACACTATCCCATATCACCAGCTCACCGTCT

TTCATTGCCATACGGAATTCCGGATGAGCATTCATCAGGCGGGCAAGAAT

GTGAATAAAGGCCGGATAAAACTTGTGCTTATTTTTCTTTACGGTCTTTA

AAAAGGCCGTAATATCCAGCTGAACGGTCTGGTTATAGGTACATTGAGCA

ACTGACTGAAATGCCTCAAAATGTTCTTTACGATGCCATTGGGATATATC

AACGGTGGTATATCCAGTGATTTTTTTCTCCATTTTAGCTTCCTTAGCTC

CTGAAAATCTCGATAACTCAAAAAATACGCCCGGTAGTGATCTTATTTCA

TTATGGTGAAAGTTGGAACCTCTTACGTGCCGATCAACGTCTCATTTTCG

CCAAAAGTTGGCCCAGGGCTTCCCGGTATCAACAGGGACACCAGGATTTA

TTTATTCTGCGAAGTGATCTTCCGTCACACTCGAGATTAATTAATCCCGC

CACCATCGTAATCCTGTTTGTTGCCCTGTACTGGTAAGGAGCCTGAGATG

ATTAATCCAAATCCAAAGCGTTCTGACGAACCGGTATTCTGGGGCCTCTT

CGGGGCCGGTGGTATGTGGAGCGCCATCATTGCGCCGGTGATGATCCTGC

TGGTGGGTATTCTGCTGCCACTGGGGTTGTTTCCGGGTGATGCGCTGAGC

TACGAGCGCGTTCTGGCGTTCGCGCAGAGCTTCATTGGTCGCGTATTCCT

GTTCCTGATGATCGTTCTGCCGCTGTGGTGTGGTTTACACCGTATGCACC

ACGCGATGCACGATCTGAAAATCCACGTACCTGCGGGCAAATGGGTTTTC

TACGGTCTGGCTGCTATCCTGACAGTTGTCACGCTGATTGGTGAAGGGCG

AATTCTGCAGATATCCATCACACTGGCGGCCGCTCGAGCATGCATCTAGA

GGGCCCAATTCGCCCTATAGTGAGTCGTATTACAATTCACTGGCCGTCGT

TTTACAACGTCGTGACTGGGAAAACCCTGGCGTTACCCAACTTAATCGCC

TTGCAGCACATCCCCCTTTCGCCAGCTGGCGTAATAGCGAAGAGGCCCGC

ACCGATCGCCCTTCCCAACAGTTGCGCAGCCTGAATGGCGAATGGACGCG

CCCTGTAGCGGCGCATTAAGCGCGGCGGGTGTGGTGGTTACGCGCAGCGT

GACCGCTACACTTGCCAGCGCCCTAGCGCCCGCTCCTTTCGCTTTCTTCC

CTTCCTTTCTCGCCACGTTCGCCGGCTTTCCCCGTCAAGCTCTAAATCGG

GGGCTCCCTTTAGGGTTCCGATTTAGTGCTTTACGGCACCTCGACCCCAA

AAAACTTGATTAGGGTGATGGTTCACGTAGTGGGCCATCGCCCTGATAGA

CGGTTTTTCGCCCTTTGACGTTGGAGTCCACGTTCTTTAATAGTGGACTC

TTGTTCCAAACTGGAACAACACTCAACCCTATCTCGGTCTATTCTTTTGA

TTTATAAGGGATTTTGCCGATTTCGGCCTATTGGTTAAAAAATGAGCTGA

TTTAACAAAAATTTAACGCGAATTTTAACAAAATTCAGGGCGCAAGGGCT

GCTAAAGGAAGCGGAACACGTAGAAAGCCAGTCCGCAGAAACGGTGCTGA

CCCCGGATGAATGTCAGCTACTGGGCTATCTGGACAAGGGAAAACGCAAG

CGCAAAGAGAAAGCAGGTAGCTTGCAGTGGGCTTACATGGCGATAGCTAG

ACTGGGCGGTTTTATGGACAGCAAGCGAACCGGAATTGCCAGCTGGGGCG

CCCTCTGGTAAGGTTGGGAAGCCCTGCAAAGTAAACTGGATGGCTTTCTT

GCCGCCAAGGATCTGATGGCGCAGGGGATCAAGATCTGATCAAGAGACAG

GATGAGGATCGTTTCGCATGATTGAACAAGATGGATTGCACGCAGGTTCT

CCGGCCGCTTGGGTGGAGAGGCTATTCGGCTATGACTGGGCACAACAGAC

AATCGGCTGCTCTGATGCCGCCGTGTTCCGGCTGTCAGCGCAGGGGCGCC

CGGTTCTTTTTGTCAAGACCGACCTGTCCGGTGCCCTGAATGAACTGCAG

GACGAGGCAGCGCGGCTATCGTGGCTGGCCACGACGGGCGTTCCTTGCGC

AGCTGTGCTCGACGTTGTCACTGAAGCGGGAAGGGACTGGCTGCTATTGG

GCGAAGTGCCGGGGCAGGATCTCCTGTCATCCCACCTTGCTCCTGCCGAG

AAAGTATCCATCATGGCTGATGCAATGCGGCGGCTGCATACGCTTGATCC

GGCTACCTGCCCATTCGACCACCAAGCGAAACATCGCATCGAGCGAGCAC

GTACTCGGATGGAAGCCGGTCTTGTCGATCAGGATGATCTGGACGAAGAG

CATCAGGGGCTCGCGCCAGCCGAACTGTTCGCCAGGCTCAAGGCGCGCAT

GCCCGACGGCGAGGATCTCGTCGTGACCCATGGCGATGCCTGCTTGCCGA

ATATCATGGTGGAAAATGGCCGCTTTTCTGGATTCATCGACTGTGGCCGG

CTGGGTGTGGCGGACCGCTATCAGGACATAGCGTTGGCTACCCGTGATAT

TGCTGAAGAGCTTGGCGGCGAATGGGCTGACCGCTTCCTCGTGCTTTACG

GTATCGCCGCTCCCGATTCGCAGCGCATCGCCTTCTATCGCCTTCTTGAC

GAGTTCTTCTGAATTGAAAAAGGAAGAGTATGAGTATTCAACATTTCCGT

GTCGCCCTTATTCCCTTTTTTGCGGCATTTTGCCTTCCTGTTTTTGCTCA

CCCAGAAACGCTGGTGAAAGTAAAAGATGCTGAAGATCAGTTGGGTGCAC

GAGTGGGTTACATCGAACTGGATCTCAACAGCGGTAAGATCCTTGAGAGT

TTTCGCCCCGAAGAACGTTTTCCAATGATGAGCACTTTTAAAGTTCTGCT

ATGTGGCGCGGTATTATCCCGTATTGACGCCGGGCAAGAGCAACTCGGTC

GCCGCATACACTATTCTCAGAATGACTTGGTTGAGTACTCACCAGTCACA

GAAAAGCATCTTACGGATGGCATGACAGTAAGAGAATTATGCAGTGCTGC

CATAACCATGAGTGATAACACTGCGGCCAACTTACTTCTGACAACGATCG

GAGGACCGAAGGAGCTAACCGCTTTTTTGCACAACATGGGGGATCATGTA

ACTCGCCTTGATCGTTGGGAACCGGAGCTGAATGAAGCCATACCAAACGA

CGAGCGTGACACCACGATGCCTGTAGCAATGGCAACAACGTTGCGCAAAC

TATTAACTGGCGAACTACTTACTCTAGCTTCCCGGCAACAATTAATAGAC

TGGATGGAGGCGGATAAAGTTGCAGGACCACTTCTGCGCTCGGCCCTTCC

GGCTGGCTGGTTTATTGCTGATAAATCTGGAGCCGGTGAGCGTGGGTCTC

GCGGTATCATTGCAGCACTGGGGCCAGATGGTAAGCCCTCCCGTATCGTA

GTTATCTACACGACGGGGAGTCAGGCAACTATGGATGAACGAAATAGACA

GATCGCTGAGATAGGTGCCTCACTGATTAAGCATTGGTAACTGTCAGACC

AAGTTTACTCATATATACTTTAGATTGATTTAAAACTTCATTTTTAATTT

AAAAGGATCTAGGTGAAGATCCTTTTTGATAATCTCATGACCAAAATCCC

TTAACGTGAGTTTTCGTTCCACTGAGCGTCAGACCCCGTAGAAAAGATCA

AAGGATCTTCTTGAGATCCTTTTTTTCTGCGCGTAATCTGCTGCTTGCAA

ACAAAAAAACCACCGCTACCAGCGGTGGTTTGTTTGCCGGATCAAGAGCT

ACCAACTCTTTTTCCGAAGGTAACTGGCTTCAGCAGAGCGCAGATACCAA

ATACTGTTCTTCTAGTGTAGCCGTAGTTAGGCCACCACTTCAAGAACTCT

GTAGCACCGCCTACATACCTCGCTCTGCTAATCCTGTTACCAGTGGCTGC

TGCCAGTGGCGATAAGTCGTGTCTTACCGGGTTGGACTCAAGACGATAGT

TACCGGATAAGGCGCAGCGGTCGGGCTGAACGGGGGGTTCGTGCACACAG

CCCAGCTTGGAGCGAACGACCTACACCGAACTGAGATACCTACAGCGTGA

GCTATGAGAAAGCGCCACGCTTCCCGAAGGGAGAAAGGCGGACAGGTATC

CGGTAAGCGGCAGGGTCGGAACAGGAGAGCGCACGAGGGAGCTTCCAGGG

GGAAACGCCTGGTATCTTTATAGTCCTGTCGGGTTTCGCCACCTCTGACT

TGAGCGTCGATTTTTGTGATGCTCGTCAGGGGGGCGGAGCCTATGGAAAA

ACGCCAGCAACGCGGCCTTTTTACGGTTCCTGGCCTTTTGCTGGCCTTTT

GCTCACATGTTCTTTCCTGCGTTATCCCCTGATTCTGTGGATAACCGTAT

TACCGCCTTTGAGTGAGCTGATACCGCTCGCCGCAGCCGAACGACCGAGC

GCAGCGAGTCAGTGAGCGAGGAAGCGGAAG

pQL006-Mmm

>pQL006-Mmm Ligation of mmm into frdB

AGCGCCCAATACGCAAACCGCCTCTCCCCGCGCGTTGGCCGATTCATTAA

TGCAGCTGGCACGACAGGTTTCCCGACTGGAAAGCGGGCAGTGAGCGCAA

CGCAATTAATGTGAGTTAGCTCACTCATTAGGCACCCCAGGCTTTACACT

TTATGCTTCCGGCTCGTATGTTGTGTGGAATTGTGAGCGGATAACAATTT

CACACAGGAAACAGCTATGACCATGATTACGCCAAGCTTGGTACCGAGCT

CGGATCCACTAGTAACGGCCGCCAGTGTGCTGGAATTCGCCCTTTGCAGA

AAACCATCGACAAGCTGGCAGAGCTGCAGGAACGCTTCAAGCGCGTGCGC

ATCACCGACACTTCCAGCGTGTTCAACACCGACCTGCTCTACACCATTGA

ACTGGGCCACGGTCTGAACGTTGCTGAATGTATGGCGCACTCCGCAATGG

CACGTAAAGAGTCCCGCGGCGCGCACCAGCGTCTGGACGAAGGTTGCACC

GAGCGTGACGACGTCAACTTCCTCAAACACACCCTCGCCTTCCGCGATGC

TGATGGCACGACTCGCCTGGAGTACAGCGACGTGAAGATTACTACGCTGC

CGCCAGCTAAACGCGTTTACGGTGGCGAAGCGGATGCAGCCGATAAGGCG

GAAGCAGCCAATAAGAAGGAGAAGGCGAATGGCTGAGATGAAAAACCTGA

AAATTGAGGTGGTGCGCTATCGGATCCAGGAGGATTACTATATGAAACTC

TCAACTAAACTTTGTTGGTGTGGTATTAAAGGAAGACTTAGGCCGCAAAA

GCAACAACAATTACACAATACAAACTTGCAAATGACTGAACTAAAAAAAC

AAAAGACCGCTGAACAAAAAACCAGACCTCAAAATGTCGGTATTAAAGGT

ATCCAAATTTACATCCCAACTCAATGTGTCAACCAATCTGAGCTAGAGAA

ATTTGATGGCGTTTCTCAAGGTAAATACACAATTGGTCTGGGCCAAACCA

ACATGTCTTTTGTCAATGACAGAGAAGATATCTACTCGATGTCCCTAACT

GTTTTGTCTAAGTTGATCAAGAGTTACAACATCGACACCAACAAAATTGG

TAGATTAGAAGTCGGTACTGAAACTCTGATTGACAAGTCCAAGTCTGTCA

AGTCTGTCTTGATGCAATTGTTTGGTGAAAACACTGACGTCGAAGGTATT

GACACGCTTAATGCCTGTTACGGTGGTACCAACGCGTTGTTCAACTCTTT

GAACTGGATTGAATCTAACGCATGGGATGGTAGAGACGCCATTGTAGTTT

GCGGTGATATTGCCATCTACGATAAGGGTGCCGCAAGACCAACCGGTGGT

GCCGGTACTGTTGCTATGTGGATCGGTCCTGATGCTCCAATTGTATTTGA

CTCTGTAAGAGCTTCTTACATGGAACACGCCTACGATTTTTACAAGCCAG

ATTTCACCAGCGAATATCCTTACGTCGATGGTCATTTTTCATTAACTTGT

TACGTCAAGGCTCTTGATCAAGTTTACAAGAGTTATTCCAAGAAGGCTAT

TTCTAAAGGGTTGGTTAGCGATCCCGCTGGTTCGGATGCTTTGAACGTTT

TGAAATATTTCGACTACAACGTTTTCCATGTTCCAACCTGTAAATTGGTC

ACAAAATCATACGGTAGATTACTATATAACGATTTCAGAGCCAATCCTCA

ATTGTTCCCAGAAGTTGACGCCGAATTAGCTACTCGCGATTATGACGAAT

CTTTAACCGATAAGAACATTGAAAAAACTTTTGTTAATGTTGCTAAGCCA

TTCCACAAAGAGAGAGTTGCCCAATCTTTGATTGTTCCAACAAACACAGG

TAACATGTACACCGCATCTGTTTATGCCGCCTTTGCATCTCTATTAAACT

ATGTTGGATCTGACGACTTACAAGGCAAGCGTGTTGGTTTATTTTCTTAC

GGTTCCGGTTTAGCTGCATCTCTATATTCTTGCAAAATTGTTGGTGACGT

CCAACATATTATCAAGGAATTAGATATTACTAACAAATTAGCCAAGAGAA

TCACCGAAACTCCAAAGGATTACGAAGCTGCCATCGAATTGAGAGAAAAT

GCCCATTTGAAGAAGAACTTCAAACCTCAAGGTTCCATTGAGCATTTGCA

AAGTGGTGTTTACTACTTGACCAACATCGATGACAAATTTAGAAGATCTT

ACGATGTTAAAAAATAGGAGGCAGATCAAATGTCAGAGTTGAGAGCCTTC

AGTGCCCCAGGGAAAGCGTTACTAGCTGGTGGATATTTAGTTTTAGATAC

AAAATATGAAGCATTTGTAGTCGGATTATCGGCAAGAATGCATGCTGTAG

CCCATCCTTACGGTTCATTGCAAGGGTCTGATAAGTTTGAAGTGCGTGTG

AAAAGTAAACAATTTAAAGATGGGGAGTGGCTGTACCATATAAGTCCTAA

AAGTGGCTTCATTCCTGTTTCGATAGGCGGATCTAAGAACCCTTTCATTG

AAAAAGTTATCGCTAACGTATTTAGCTACTTTAAACCTAACATGGACGAC

TACTGCAATAGAAACTTGTTCGTTATTGATATTTTCTCTGATGATGCCTA

CCATTCTCAGGAGGATAGCGTTACCGAACATCGTGGCAACAGAAGATTGA

GTTTTCATTCGCACAGAATTGAAGAAGTTCCCAAAACAGGGCTGGGCTCC

TCGGCAGGTTTAGTCACAGTTTTAACTACAGCTTTGGCCTCCTTTTTTGT

ATCGGACCTGGAAAATAATGTAGACAAATATAGAGAAGTTATTCATAATT

TAGCACAAGTTGCTCATTGTCAAGCTCAGGGTAAAATTGGAAGCGGGTTT

GATGTAGCGGCGGCAGCATATGGATCTATCAGATATAGAAGATTCCCACC

CGCATTAATCTCTAATTTGCCAGATATTGGAAGTGCTACTTACGGCAGTA

AACTGGCGCATTTGGTTGATGAAGAAGACTGGAATATTACGATTAAAAGT

AACCATTTACCTTCGGGATTAACTTTATGGATGGGCGATATTAAGAATGG

TTCAGAAACAGTAAAACTGGTCCAGAAGGTAAAAAATTGGTATGATTCGC

ATATGCCAGAAAGCTTGAAAATATATACAGAACTCGATCATGCAAATTCT

AGATTTATGGATGGACTATCTAAACTAGATCGCTTACACGAGACTCATGA

CGATTACAGCGATCAGATATTTGAGTCTCTTGAGAGGAATGACTGTACCT

GTCAAAAGTATCCTGAAATCACAGAAGTTAGAGATGCAGTTGCCACAATT

AGACGTTCCTTTAGAAAAATAACTAAAGAATCTGGTGCCGATATCGAACC

TCCCGTACAAACTAGCTTATTGGATGATTGCCAGACCTTAAAAGGAGTTC

TTACTTGCTTAATACCTGGTGCTGGTGGTTATGACGCCATTGCAGTGATT

ACTAAGCAAGATGTTGATCTTAGGGCTCAAACCGCTAATGACAAAAGATT

TTCTAAGGTTCAATGGCTGGATGTAACTCAGGCTGACTGGGGTGTTAGGA

AAGAAAAAGATCCGGAAACTTATCTTGATAAATAGGAGGTAATACTCATG

ACCGTTTACACAGCATCCGTTACCGCACCCGTCAACATCGCAACCCTTAA

GTATTGGGGGAAAAGGGACACGAAGTTGAATCTGCCCACCAATTCGTCCA

TATCAGTGACTTTATCGCAAGATGACCTCAGAACGTTGACCTCTGCGGCT

ACTGCACCTGAGTTTGAACGCGACACTTTGTGGTTAAATGGAGAACCACA

CAGCATCGACAATGAAAGAACTCAAAATTGTCTGCGCGACCTACGCCAAT

TAAGAAAGGAAATGGAATCGAAGGACGCCTCATTGCCCACATTATCTCAA

TGGAAACTCCACATTGTCTCCGAAAATAACTTTCCTACAGCAGCTGGTTT

AGCTTCCTCCGCTGCTGGCTTTGCTGCATTGGTCTCTGCAATTGCTAAGT

TATACCAATTACCACAGTCAACTTCAGAAATATCTAGAATAGCAAGAAAG

GGGTCTGGTTCAGCTTGTAGATCGTTGTTTGGCGGATACGTGGCCTGGGA

AATGGGAAAAGCTGAAGATGGTCATGATTCCATGGCAGTACAAATCGCAG

ACAGCTCTGACTGGCCTCAGATGAAAGCTTGTGTCCTAGTTGTCAGCGAT

ATTAAAAAGGATGTGAGTTCCACTCAGGGTATGCAATTGACCGTGGCAAC

CTCCGAACTATTTAAAGAAAGAATTGAACATGTCGTACCAAAGAGATTTG

AAGTCATGCGTAAAGCCATTGTTGAAAAAGATTTCGCCACCTTTGCAAAG

GAAACAATGATGGATTCCAACTCTTTCCATGCCACATGTTTGGACTCTTT

CCCTCCAATATTCTACATGAATGACACTTCCAAGCGTATCATCAGTTGGT

GCCACACCATTAATCAGTTTTACGGAGAAACAATCGTTGCATACACGTTT

GATGCAGGTCCAAATGCTGTGTTGTACTACTTAGCTGAAAATGAGTCGAA

ACTCTTTGCATTTATCTATAAATTGTTTGGCTCTGTTCCTGGATGGGACA

AGAAATTTACTACTGAGCAGCTTGAGGCTTTCAACCATCAATTTGAATCA

TCTAACTTTACTGCACGTGAATTGGATCTTGAGTTGCAAAAGGATGTTGC

CAGAGTGATTTTAACTCAAGTCGGTTCAGGCCCACAAGAAACAAACGAAT

CTTTGATTGACGCAAAGACTGGTCTACCAAAGGAATAAGTCGACCTGCAG

GCATGCAAGCTTGGCTGTTTTGGCGGATGAGAGAAGATTTTCAGCCTGAT

ACAGATTAAATCAGAACGCAGAAGCGGTCTGATAAAACAGAATTTGCCTG

GCGGCAGTAGCGCGGTGGTCCCACCTGACCCCATGCCGAACTCAGAAGTG

AAACGCCGTAGCGCCGATGGTAGTGTGGGGTCTCCCCATGCGAGAGTAGG

GAACTGCCAGGCATCAAATAAAACGAAAGGCTCAGTCGAAAGACTGGGCC

TTTCGTTTTATCTGTTGTTTGTCGGTGAACGCTCTCCTGAGTAGGACAAA

TCCGCCGGGAGCGGATTTGAACGTTGCGAAGCAACGGCCCGGAGGGTGGC

GGGCAGGACGCCCGCCATAAACTGCCAGGCATCAAATTAAGCAGAAGGCC

ATCCTGACGGATGGCCTTTTTGCGTTTCTGCCACCATCGTAATCCTGTTT

GTTGCCCTGTACTGGTAAGGAGCCTGAGATGATTAATCCAAATCCAAAGC

GTTCTGACGAACCGGTATTCTGGGGCCTCTTCGGGGCCGGTGGTATGTGG

AGCGCCATCATTGCGCCGGTGATGATCCTGCTGGTGGGTATTCTGCTGCC

ACTGGGGTTGTTTCCGGGTGATGCGCTGAGCTACGAGCGCGTTCTGGCGT

TCGCGCAGAGCTTCATTGGTCGCGTATTCCTGTTCCTGATGATCGTTCTG

CCGCTGTGGTGTGGTTTACACCGTATGCACCACGCGATGCACGATCTGAA

AATCCACGTACCTGCGGGCAAATGGGTTTTCTACGGTCTGGCTGCTATCC

TGACAGTTGTCACGCTGATTGGTGAAGGGCGAATTCTGCAGATATCCATC

ACACTGGCGGCCGCTCGAGCATGCATCTAGAGGGCCCAATTCGCCCTATA

GTGAGTCGTATTACAATTCACTGGCCGTCGTTTTACAACGTCGTGACTGG

GAAAACCCTGGCGTTACCCAACTTAATCGCCTTGCAGCACATCCCCCTTT

CGCCAGCTGGCGTAATAGCGAAGAGGCCCGCACCGATCGCCCTTCCCAAC

AGTTGCGCAGCCTGAATGGCGAATGGACGCGCCCTGTAGCGGCGCATTAA

GCGCGGCGGGTGTGGTGGTTACGCGCAGCGTGACCGCTACACTTGCCAGC

GCCCTAGCGCCCGCTCCTTTCGCTTTCTTCCCTTCCTTTCTCGCCACGTT

CGCCGGCTTTCCCCGTCAAGCTCTAAATCGGGGGCTCCCTTTAGGGTTCC

GATTTAGTGCTTTACGGCACCTCGACCCCAAAAAACTTGATTAGGGTGAT

GGTTCACGTAGTGGGCCATCGCCCTGATAGACGGTTTTTCGCCCTTTGAC

GTTGGAGTCCACGTTCTTTAATAGTGGACTCTTGTTCCAAACTGGAACAA

CACTCAACCCTATCTCGGTCTATTCTTTTGATTTATAAGGGATTTTGCCG

ATTTCGGCCTATTGGTTAAAAAATGAGCTGATTTAACAAAAATTTAACGC

GAATTTTAACAAAATTCAGGGCGCAAGGGCTGCTAAAGGAAGCGGAACAC

GTAGAAAGCCAGTCCGCAGAAACGGTGCTGACCCCGGATGAATGTCAGCT

ACTGGGCTATCTGGACAAGGGAAAACGCAAGCGCAAAGAGAAAGCAGGTA

GCTTGCAGTGGGCTTACATGGCGATAGCTAGACTGGGCGGTTTTATGGAC

AGCAAGCGAACCGGAATTGCCAGCTGGGGCGCCCTCTGGTAAGGTTGGGA

AGCCCTGCAAAGTAAACTGGATGGCTTTCTTGCCGCCAAGGATCTGATGG

CGCAGGGGATCAAGATCTGATCAAGAGACAGGATGAGGATCGTTTCGCAT

GATTGAACAAGATGGATTGCACGCAGGTTCTCCGGCCGCTTGGGTGGAGA

GGCTATTCGGCTATGACTGGGCACAACAGACAATCGGCTGCTCTGATGCC

GCCGTGTTCCGGCTGTCAGCGCAGGGGCGCCCGGTTCTTTTTGTCAAGAC

CGACCTGTCCGGTGCCCTGAATGAACTGCAGGACGAGGCAGCGCGGCTAT

CGTGGCTGGCCACGACGGGCGTTCCTTGCGCAGCTGTGCTCGACGTTGTC

ACTGAAGCGGGAAGGGACTGGCTGCTATTGGGCGAAGTGCCGGGGCAGGA

TCTCCTGTCATCCCACCTTGCTCCTGCCGAGAAAGTATCCATCATGGCTG

ATGCAATGCGGCGGCTGCATACGCTTGATCCGGCTACCTGCCCATTCGAC

CACCAAGCGAAACATCGCATCGAGCGAGCACGTACTCGGATGGAAGCCGG

TCTTGTCGATCAGGATGATCTGGACGAAGAGCATCAGGGGCTCGCGCCAG

CCGAACTGTTCGCCAGGCTCAAGGCGCGCATGCCCGACGGCGAGGATCTC

GTCGTGACCCATGGCGATGCCTGCTTGCCGAATATCATGGTGGAAAATGG

CCGCTTTTCTGGATTCATCGACTGTGGCCGGCTGGGTGTGGCGGACCGCT

ATCAGGACATAGCGTTGGCTACCCGTGATATTGCTGAAGAGCTTGGCGGC

GAATGGGCTGACCGCTTCCTCGTGCTTTACGGTATCGCCGCTCCCGATTC

GCAGCGCATCGCCTTCTATCGCCTTCTTGACGAGTTCTTCTGAATTGAAA

AAGGAAGAGTATGAGTATTCAACATTTCCGTGTCGCCCTTATTCCCTTTT

TTGCGGCATTTTGCCTTCCTGTTTTTGCTCACCCAGAAACGCTGGTGAAA

GTAAAAGATGCTGAAGATCAGTTGGGTGCACGAGTGGGTTACATCGAACT

GGATCTCAACAGCGGTAAGATCCTTGAGAGTTTTCGCCCCGAAGAACGTT

TTCCAATGATGAGCACTTTTAAAGTTCTGCTATGTGGCGCGGTATTATCC

CGTATTGACGCCGGGCAAGAGCAACTCGGTCGCCGCATACACTATTCTCA

GAATGACTTGGTTGAGTACTCACCAGTCACAGAAAAGCATCTTACGGATG

GCATGACAGTAAGAGAATTATGCAGTGCTGCCATAACCATGAGTGATAAC

ACTGCGGCCAACTTACTTCTGACAACGATCGGAGGACCGAAGGAGCTAAC

CGCTTTTTTGCACAACATGGGGGATCATGTAACTCGCCTTGATCGTTGGG

AACCGGAGCTGAATGAAGCCATACCAAACGACGAGCGTGACACCACGATG

CCTGTAGCAATGGCAACAACGTTGCGCAAACTATTAACTGGCGAACTACT

TACTCTAGCTTCCCGGCAACAATTAATAGACTGGATGGAGGCGGATAAAG

TTGCAGGACCACTTCTGCGCTCGGCCCTTCCGGCTGGCTGGTTTATTGCT

GATAAATCTGGAGCCGGTGAGCGTGGGTCTCGCGGTATCATTGCAGCACT

GGGGCCAGATGGTAAGCCCTCCCGTATCGTAGTTATCTACACGACGGGGA

GTCAGGCAACTATGGATGAACGAAATAGACAGATCGCTGAGATAGGTGCC

TCACTGATTAAGCATTGGTAACTGTCAGACCAAGTTTACTCATATATACT

TTAGATTGATTTAAAACTTCATTTTTAATTTAAAAGGATCTAGGTGAAGA

TCCTTTTTGATAATCTCATGACCAAAATCCCTTAACGTGAGTTTTCGTTC

CACTGAGCGTCAGACCCCGTAGAAAAGATCAAAGGATCTTCTTGAGATCC

TTTTTTTCTGCGCGTAATCTGCTGCTTGCAAACAAAAAAACCACCGCTAC

CAGCGGTGGTTTGTTTGCCGGATCAAGAGCTACCAACTCTTTTTCCGAAG

GTAACTGGCTTCAGCAGAGCGCAGATACCAAATACTGTTCTTCTAGTGTA

GCCGTAGTTAGGCCACCACTTCAAGAACTCTGTAGCACCGCCTACATACC

TCGCTCTGCTAATCCTGTTACCAGTGGCTGCTGCCAGTGGCGATAAGTCG

TGTCTTACCGGGTTGGACTCAAGACGATAGTTACCGGATAAGGCGCAGCG

GTCGGGCTGAACGGGGGGTTCGTGCACACAGCCCAGCTTGGAGCGAACGA

CCTACACCGAACTGAGATACCTACAGCGTGAGCTATGAGAAAGCGCCACG

CTTCCCGAAGGGAGAAAGGCGGACAGGTATCCGGTAAGCGGCAGGGTCGG

AACAGGAGAGCGCACGAGGGAGCTTCCAGGGGGAAACGCCTGGTATCTTT

ATAGTCCTGTCGGGTTTCGCCACCTCTGACTTGAGCGTCGATTTTTGTGA

TGCTCGTCAGGGGGGCGGAGCCTATGGAAAAACGCCAGCAACGCGGCCTT

TTTACGGTTCCTGGCCTTTTGCTGGCCTTTTGCTCACATGTTCTTTCCTG

CGTTATCCCCTGATTCTGTGGATAACCGTATTACCGCCTTTGAGTGAGCT

GATACCGCTCGCCGCAGCCGAACGACCGAGCGCAGCGAGTCAGTGAGCGA

GGAAGCGGAAG
